# Light on Alzheimer’s disease: from basic insights to preclinical studies

**DOI:** 10.3389/fnagi.2024.1363458

**Published:** 2024-03-18

**Authors:** Jie Mi, Chao Liu, Honglei Chen, Yan Qian, Jingyi Zhu, Yachao Zhang, Yizhi Liang, Lidai Wang, Dean Ta

**Affiliations:** ^1^Yiwu Research Institute, Fudan University, Yiwu, China; ^2^Digital Medical Research Center, School of Basic Medical Sciences, Fudan University, Shanghai, China; ^3^Shanghai Key Laboratory of Medical Imaging Computing and Computer Assisted Intervention, Shanghai, China; ^4^Department of Biomedical Engineering, City University of Hong Kong, Kowloon, Hong Kong SAR, China; ^5^Medical Ultrasound Department, Suzhou Institute of Biomedical Engineering and Technology, Chinese Academy of Sciences, Suzhou, China; ^6^Guangdong Provincial Key Laboratory of Optical Fiber Sensing and Communications, Institute of Photonics Technology, Jinan University, Guangzhou, China; ^7^Department of Electronic Engineering, Fudan University, Shanghai, China

**Keywords:** Alzheimer’s disease, brain imaging, imaging modalities, photoacoustic imaging, diagnose and theranostics

## Abstract

Alzheimer’s disease (AD), referring to a gradual deterioration in cognitive function, including memory loss and impaired thinking skills, has emerged as a substantial worldwide challenge with profound social and economic implications. As the prevalence of AD continues to rise and the population ages, there is an imperative demand for innovative imaging techniques to help improve our understanding of these complex conditions. Photoacoustic (PA) imaging forms a hybrid imaging modality by integrating the high-contrast of optical imaging and deep-penetration of ultrasound imaging. PA imaging enables the visualization and characterization of tissue structures and multifunctional information at high resolution and, has demonstrated promising preliminary results in the study and diagnosis of AD. This review endeavors to offer a thorough overview of the current applications and potential of PA imaging on AD diagnosis and treatment. Firstly, the structural, functional, molecular parameter changes associated with AD-related brain imaging captured by PA imaging will be summarized, shaping the diagnostic standpoint of this review. Then, the therapeutic methods aimed at AD is discussed further. Lastly, the potential solutions and clinical applications to expand the extent of PA imaging into deeper AD scenarios is proposed. While certain aspects might not be fully covered, this mini-review provides valuable insights into AD diagnosis and treatment through the utilization of innovative tissue photothermal effects. We hope that it will spark further exploration in this field, fostering improved and earlier theranostics for AD.

## 1 Introduction

Alzheimer’s disease (AD), typically embodied by dysmnesia, language deficits, cognitive bewilderment and behavioral anomalies, is a progressive neurodegenerative condition that unfolds gradually, worsening over time and irreversibly diminishing the patient’s ability for self-care (Hong and Yaqub, 2019; Scheltens et al., 2021). AD, like cancer, heart disease, and cerebrovascular disease, stands as a major cause of mortality among the elderly (Sperling et al., 2013; Liu K.Y. et al., 2021). As the aging population grows more pronounced, AD could emerge as one of the most costly diseases for society, imposing a significant burden on the economy. The data from 2015 reveals that approximately 29.8 million individuals globally experienced the effects of AD, and this number is anticipated to increase with the continuous aging of the population (Vos et al., 2016). Commonly, the progression of AD can be delineated into four phases: preclinical AD, early stage, middle stage, and late stage. The initial phase, often termed mild cognitive impairment (MCI) (Arnáiz and Almkvist, 2003), signifies a transitional period from normality to memory loss. Subsequently, individuals with AD may exhibit escalating difficulties in learning and memory, eventually leading to a conclusive diagnosis by healthcare professionals. As the ailment advances, patients face challenges in living independently and carrying out routine daily tasks (Förstl and Kurz, 1999). Finally, patients reach a stage of complete dependency on caregivers (Förstl and Kurz, 1999). The fundamental pathological characteristics of AD, as indicated by relevant research, mainly represented by the accumulation of amyloid-β (Aβ) plaques and hyperphosphorylation of tau (tubulin-associated unit) proteins, and the former primarily occurs in the hippocampus and cortex of the brain, while the latter mainly leads to the formation of neurofibrillary tangles (NFTs) (Serrano-Pozo et al., 2011; Ge et al., 2021). These changes might manifest years before symptoms emerge. Additionally, the substantial presence of lipid inclusions ascertained from AD patients suggests that lipid peroxidation signifies an early stage in AD development (Ayala et al., 2014; Kao et al., 2020). Hence, monitoring lipid content becomes imperative for early AD diagnosis (Salinas et al., 2022). As clinical trials gather more data on AD-related risk factors, there’s an increasing emphasis on early diagnosis and treatment for AD patients. Delayed intervention stands as the primary cause of treatment failure in this context (Sperling et al., 2011; Teipel et al., 2015).

Brain imaging technology has profoundly influenced the acquisition of information concerning brain structure and function, significantly impacting clinical applications. It has become pivotal in identifying preventive treatments and AD-modifying interventions. Biomarkers found in bodily fluids (such as cerebrospinal fluid and blood) and imaging exert a pivotal role as chemical indicators in assessing the risk of brain diseases (Teipel et al., 2015). Various biomarkers reflect distinct physiological information and demand suitable detection methods. Current imaging methodologies, including positron emission tomography (PET) and magnetic resonance imaging (MRI), which have relatively high treatment expenses, and optical coherence tomography (OCT) with certain radiation, and non-invasive ultrasound (US), are restricted to providing structural details but struggle to offer multifunctional cerebral imaging for detecting functional impairments. The capabilities of these imaging techniques are outlined in Table 1.

**Table 1:** Summary of current imaging modalities’ capacities for AD diagnosis.

Imaging modality	Capacities	Advantages	Drawbacks
PET	Feedback the brain glucose metabolism rate in the resting state (Mosconi, 2013); image Aβ deposited in the brain (Kantarci, 2014).	Functional imaging Whole-body imaging High sensitivity	Limited spatial resolution Radiation exposure
MRI	Provide high spatial resolution and sufficient contrast images and brain structural information for monitoring AD (Dickerson et al., 2009; Jack et al., 2009, 2010).	Soft tissue contrast Multi-planar imaging	Cost and accessibility Metallic implant
OCT	Provide high resolution; distinguish AD from normal aging (Kromer et al., 2014; Cunha et al., 2016).	High resolution Non-invasive High imaging speed	Limited depth penetration Few functional parameters
US	Obtain structural information from tissue echoes; achieve *in vivo* transcranial imaging (Errico et al., 2015, 2016).	Portable Inexpensive No ionizing radiation	Limited tissue contrast Few functional parameters
PA	Provide high contrast and spatial resolution with multi-functional information for vascular imaging.	High resolution High contrast Functional imaging Molecular imaging	Equipment complexity Clinical challenges

PET imaging serves as a vital tool for tumor imaging (Subramaniam et al., 2009; Davison et al., 2011; Dibble et al., 2012; Romesser et al., 2012; Agarwal et al., 2013; Davison et al., 2013; Heydarheydari et al., 2023; Lei et al., 2023; Xing et al., 2023) and is also an advanced diagnostic technique for brain imaging. It has the capability to offer details about the cerebral glucose metabolism rate during periods of rest, serving as an indicator of neural activity. Several research studies have illustrated that unique patterns of cerebral glucose metabolism can reliably distinguish AD from other disorders leading to dementia (Mosconi, 2013; Kantarci, 2014; Insel et al., 2023; Yoon et al., 2023). Furthermore, PET imaging, utilizing specific tracers, can visualize the cerebral Aβ deposition, assisting in distinguishing dementia syndromes (Kantarci, 2014). However, PET still has limitations, such as ionizing radiation, low temporal resolution (about 5–10 s), and expensive equipments.

MRI presents alluring benefits over PET imaging, including high spatial resolution and substantial contrast (Hu et al., 2022), with structural MRI being the most extensively employed method for imaging the AD brain. The structural alterations observed in clinical AD patients encompass substantial decreases in hippocampal and entorhinal cortex volume, gray matter, and cortical thickness, along with notable increases in ventricular and sulcal volume. Additionally, there are diminishments in various cerebral areas, such as the precuneus, posterior cingulate, parietal, and temporal cortex, with these alterations advancing at an expedited rate as time elapses (Dickerson et al., 2009; Jack et al., 2009, 2010). Despite its high resolution, MRI faces limitations due to its costliness for diagnosis and challenges related to patient movement. Additionally, even the fMRI, which offers the highest resolution, is unable to visualize single blood vessel (Tang et al., 2020).

Additionally, OCT and two-photon microscopy (TPM) are utilized for AD detection due to their high resolution capabilities. Taking OCT as an example, AD and typical aging processes can be distinguished by assessing the thickness of nerve fibers surrounding the optic disc, the thickness of the macula, and various vascular parameters of the retina (Kromer et al., 2014; Cunha et al., 2016; Kao et al., 2023; Ma et al., 2023). TPM enables high-resolution direct imaging of Aβ (Subramanian et al., 2020). However, both of these two pure optical imaging modalities can barely provide *in vivo* AD imaging with sufficient penetration depth due to the optical diffusion limit (~1 mm) (Liebscher and Meyer-Luehmann, 2012; Kim et al., 2016; Yan et al., 2024).

US imaging is a non-invasive imaging technique capable of capturing structural details through tissue echoes, providing an imaging depth of more than 10 cm. Through specific pulse sequences and imaging algorithms, *in vivo* high-level transcranial insight can be achieved via US imaging (Errico et al., 2015, 2016; Demené et al., 2021; Mozaffarzadeh et al., 2022). Moreover, the US can be utilized to identify modifiable risk factors linked to the progression of AD. For example, in the study by Tromso et al., atherosclerotic burden showed associations with diminished cognition and subsequent cognitive decline (Arntzen et al., 2012; Al Hazzouri et al., 2015). Karakatsani et al. found that focused ultrasound can reduce pathology and improve spatial memory in AD mice and patients (Karakatsani et al., 2023). In cardiac and vascular surgery, the detrimental effects of microscopic emboli on cognition have been firmly established. This impact is assessed using transcranial Doppler ultrasound (TCD) (Russell and Bornstein, 2005; Gaudet et al., 2010; Gasparovic et al., 2013; Yan et al., 2022). Furthermore, the blood–brain barrier (BBB) in brain can be unfolded by exploiting the microbubble contrast agents during focused ultrasound imaging processes (Li B. et al., 2023; Li D. et al., 2023). This mechanism allows for neuroimmune modulation and controlled drug release, presenting a promising therapeutic avenue for AD (Silva et al., 2018).

A similar non-invasive imaging method taking use of acoustic information is known as photoacoustic (PA) imaging, which combines the high-contrast property from optical imaging and the high-resolved characteristic from US imaging, breaking the dilemma of having to choose between imaging resolution and imaging depth for *in vivo* cerebral imaging (Beard, 2011). Based on PA, various *in vivo* applications have been conducted including liquid viscosity measurement (Zhou et al., 2021), tumor visualization, blood flow velocity measurement (Liu et al., 2020), and blood oxygen saturation (sO_2_) measurement (Liang et al., 2017; Liu C. et al., 2019; Zhu et al., 2021; Liu and Wang, 2022; Zhang et al., 2023). In PA imaging, the pulsed laser beams are used to irradiate the medium, making the endogenous chromophores absorb the incident photons. This absorption results in a brief temperature rise, subsequently causing a rising local pressure and the generation of low-amplitude ultrasonic waves with a broadband spectrum (typically tens of MHz) (Beard, 2011). The ultrasonic waves, carrying both morphological and functional information of the target, are detected by the US transducer or continuous-wave (CW) lasers (Liang et al., 2021; Liu et al., 2023), facilitating the creation of high-resolution images (Wang and Gao, 2014). Specifically, CW laser detection can be described as that by measuring the optical phase changes caused by ultrasound vibration, the resonance spectrum of local horizontal vibration can be read out. Liang et al. introduced photothermally induced acoustic vibration (PTAV) to achieve high-performance fiber PA sensing. Experimental results show that this method can provide a sub-acoustic-wavelength resolution of 10 μm, and a visualization frame rate of 50 Hz. Liu et al. used 1,550 nm CW laser as the interrogation light to detect the vibration of the Fabry–Perot (FP) cavity caused by the cavitation effect, thereby constructing a fiber optic ultrasonic endoscopic imaging probe with lateral and axial resolutions of 86 μm and 91 μm, respectively. What’s more, like US imaging, PA signal can be used to reconstruct images by special imaging algorithms (Qu et al., 2022). These waves typically encounter reduced scattering and attenuation in soft tissues compared to photons, making high-resolution, label-free PA imaging highly valuable for studying neuronal activity and hemodynamics in research settings (Hu and Wang, 2010). Specifically, Hu et al. imaged the Aβ plaques in transgenic AD mice via optical-resolution PA microscopy (OR-PAM) (Hu et al., 2009). Besides, Guo et al. introduced an arched-scanning PA microscopy (AS-PAM), which not only obtained high-resolution images of the cerebral cortex microvasculature but also analyzed information related to brain function, offering novel perspectives for neurovascular studies in the brain (Guo et al., 2023). Crucially, the diverse array of endogenous and exogenous contrast agents enables PA imaging to conduct precise and functional imaging of the brain. This capability extends to capturing both physiological and pathological information, shedding light on the stage of diseases within the brain (Wu et al., 2014). Particularly, intrinsic chromophores like oxyhemoglobin (HbO_2_) (Bohndiek et al., 2015), deoxyhemoglobin (Hb) (Yao et al., 2011, 2013; Luke and Emelianov, 2014), melanin (Strohm et al., 2013), and fat (Guggenheim et al., 2015), have different optical absorptions leading to their different contrast in PA images, such as vascularization in tumors and lipid buildup in atherosclerosis (Karande et al., 2016; Cui et al., 2017), and pigment accumulation in the skin (Strohm et al., 2013). And last but not least, there are even more types of exogenous PA contrast agents, including natural colorants (Chatni et al., 2012; Zhang et al., 2014; Zhong and Yang, 2014), nanoparticles (Jin et al., 2010; Lovell et al., 2011; Wilson et al., 2012; Bai et al., 2015; Tang et al., 2015), and reporter genes (Razansky et al., 2009; Jathoul et al., 2015; Yao and Wang, 2018), opening up enormous possibilities for diagnosing various diseases.

**Figure 1:**
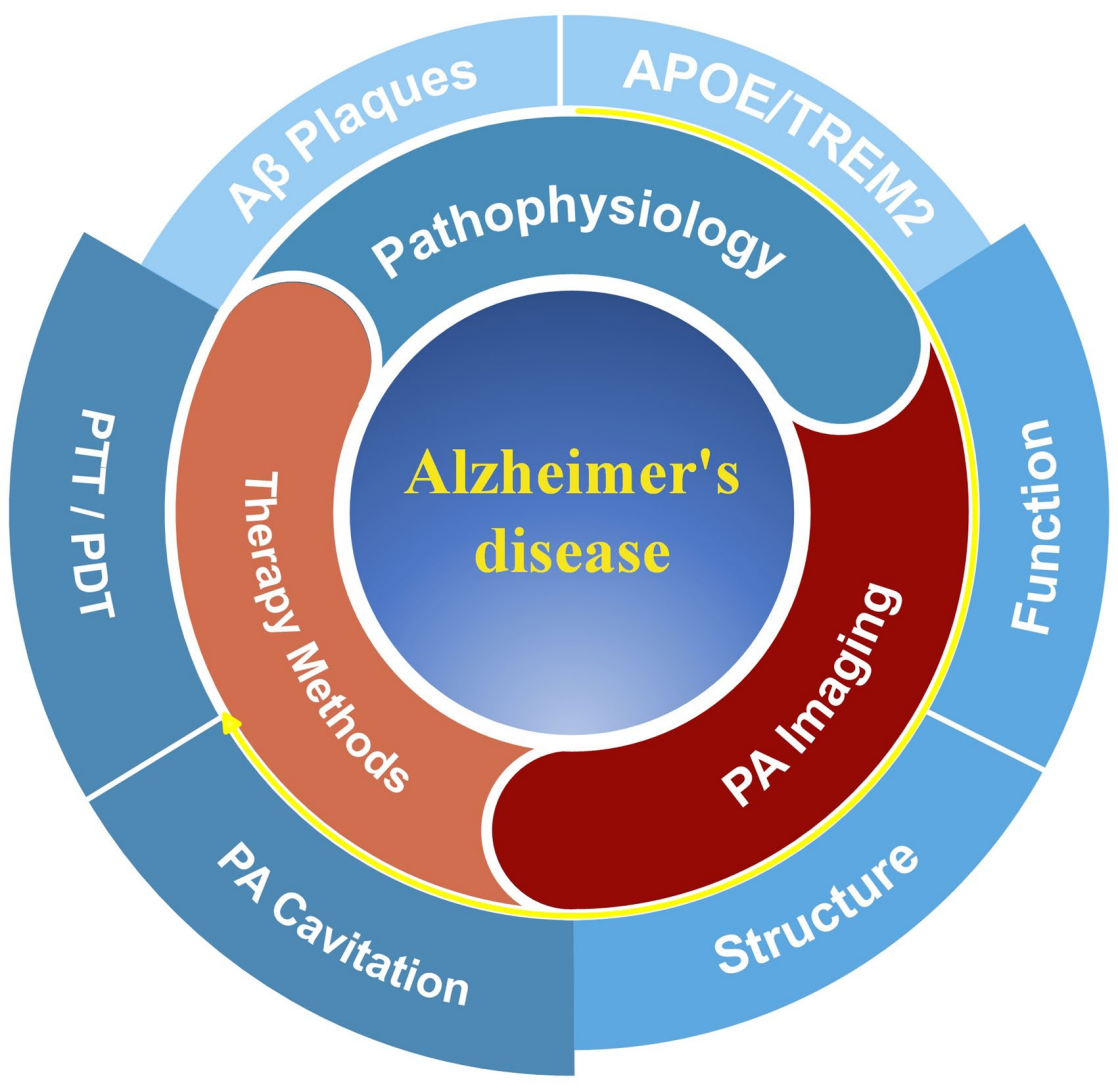
The framework of this review focuses on Alzheimer’s disease (AD) research. The typical pathologies of AD include Aβ protein deposition and risk genes (APOE and TREM2), which may induce changes in the brain structure and function of patients. These symptoms can be diagnosed through methods based on photoacoustic (PA) imaging. Additionally, there have been related research studies exploring treatments for AD, such as photodynamic therapy (PDT), photothermal therapy (PTT), and PA cavitation therapy.

The underlying goal of this review is to offer a comprehensive overview of the current applications and potential of PA technology research in AD imaging and treatment: First, the review summarizes changes in structural, functional, and molecular parameters linked to aging-related brain imaging captured by PA imaging, shaping the diagnostic perspective of the study; Then, the multimodal capabilities of PA imaging with other imaging modalities to provide a comprehensive assessment of structural and functional changes in AD are discussed; Finally, methods and technologies for PA therapy of AD are proposed (Figure 1). Although not exhaustive in some aspects, this mini-review provides a new summary and perspective on the diagnosis and treatment of AD, incorporating innovations in PA techeniques. It has the potential to inspire further explorations toward the early diagnosis and efficacious treatment of AD.

## 2 Pathophysiology of AD

AD is regarded as one of the most common types of dementia, and its main symptoms are progressive memory impairment and irreversible cognitive dysfunction (Iadanza et al., 2018). Structural changes in the AD brain are characterized by neuronal and synaptic loss in the cerebral cortex and specific subcortical areas, leading to macroanatomical atrophy due to excessive neuronal loss in some regions. Commonly impacted regions encompass the temporal and parietal lobes, segments of the frontal lobes, and the cingulate gyrus (Wenk, 2003). Several investigations employing MRI and PET have reported the atrophy of specific brain regions during AD, which can also be observed when compared to other healthy older adults (Desikan et al., 2009). The preclinical phase of AD is also referred to as the cellular stage. by basic scientists because of the changes in neurons, microglia, and astrocytes that occur during the undiscovered stage of AD (De Strooper and Karran, 2016). At the clinical stage, the development of AD is a continuous process that can evolve From regular cognitive function to cognitive decline and eventual dementia., often spanning several years. One of the most striking structural alterations in AD is Accumulation of Aβ plaques. Additionally, AD can induce neuroinflammation (Venegas et al., 2017), vascular changes (Da Mesquita et al., 2018; Sweeney et al., 2018), aging (Lu et al., 2014), and glymphatic system dysfunction (Plog and Nedergaard, 2018). Furthermore, Aβ can also induce the proliferation of tau pathological cells (Long and Holtzman, 2019), which is related to the generation of necroptosis biomarkers in brain neurons with granulovacuolar degeneration (Koper et al., 2020). From a genetic perspective, APOE and TREM2 are regarded as two major AD risk genes. Particularly, APOE commonly binds to Aβ plaques (Hansen et al., 2018), while TREM2 genetic variants associated with AD (Arg47His, Arg62His, and Asp87Asn) reduce TREM2 binding to APOE (Yeh et al., 2016).

## 3 Photoacoustic imaging in AD

PA imaging has great potential of researching the brain diseases and cancers. Initially, when the pulsed laser excites endogenous chromophores like hemoglobin and lipids, as well as external contrast agent, The photon energy absorbed leads to thermal conversion due to the photothermal effect, which causes a transient increase in temperature within the stimulated area, resulting in pressure fluctuations that generate acoustic wave signals, known as PA signals. These signals are captured by ultrasonic transducers (USTs) and then processed using various reconstruction algorithms to ultimately produce high-resolution PA images. Currently, this review main focused on and discussed the applications of PA imaging in the diagnosis of AD in small animals.

### 3.1 Conventional PA imaging systems

**Figure 2:**
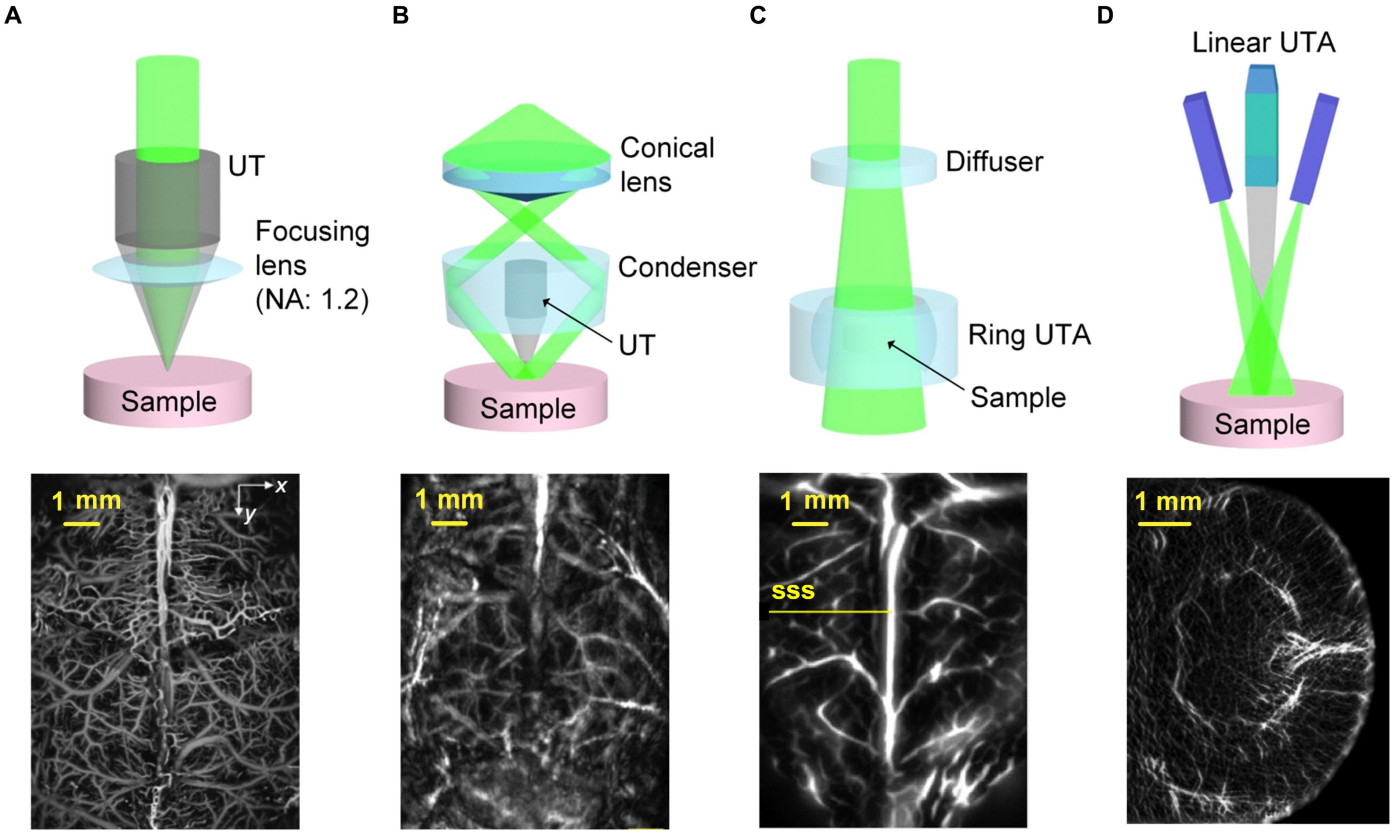
Examples illustrating PAT implementations. **(A)** The OR-PAM system observes blood vessels in the cerebral cortex of mice without damaging the skull. Reprinted with permission from Yao et al. (2015). Copyright 2015 Springer Nature. **(B)** The AR-PAM system observes blood vessels in the cerebral cortex of living mice without damaging the scalp and skull. Reprinted with permission from Yao and Wang (2013). Copyright 2013 Wiley-VCH. **(C)** Observation of cerebral cortical blood vessels using the PACT system with ring-shaped ultrasonic transducer array (UTA). SSS, superior sagittal sinus. Reprinted with permission from Li L. et al. (2017). Copyright 2017 Springer Nature. **(D)** Observation of coronal plane of a mouse brain using the PACT system with linear-shaped UTA. Reprinted with permission from Li et al. (2018a). Copyright 2018 Wiley-VCH.

According to the laser irradiation forms and ultrasonic detection ways, photoacoustic imaging systems can be roughly divided into two categories. One is PA microscopy (PAM), which is known for its high resolution, and the other is PA computed tomography (PACT), which is characterized by deep imaging (Li et al., 2021). Figure 2 displays the characteristic schematics of PA systems, accompanied by illustrative label-free cerebral vascular images acquired by using each approach (Wang and Yao, 2016). PAM relies on point by point scanning with either a tightly or weakly focused beam, while utilizing a single-element US transducer to capture the PA signals containing essential imaging details (Wang, 2009; Xia J et al., 2014). Although the PAM typically achieves a penetration depth of just a few millimeters, its spatial resolution can reach submicrons (Wang, 2009).

Based on the level of focusing, PAM can be futher divided into optically and acoustically resolved PAM, which are known as OR-PAM and AR-PAM. OR-PAM utilizes tightly focused light, forming a spot smaller than the ultrasonic focus, determining the lateral resolution. However, its depth of penetration is constrained to approximately 1 mm within soft tissue (Yao and Wang, 2013). Yao et al. used an OR-PAM system to image the mouse cerebral cortex (Yao et al., 2015), whose lateral and axial resolutions were 3 μm and 15 μm, respectively. And the maximum imaging depth could reach 0.7 mm with a fast-scanning ability that the acquisition time is only about 15 s when imaged the region at the size of 5 × 10 mm^2^ (Figure 2A). AR-PAM, characterized by the combination of loosely focused laser and highly focused US transducer bring about a relatively high resolution in the quasi-diffusive regime (Wang and Wu, 2012; Yao and Wang, 2013). Uniform light illuminates the region of interest, generating PA signals that are captured by the array transducer, and images are reconstructed using time-of-flight (ToF) based inversion algorithms (Xia J et al., 2014). Yao and Wang used an AR-PAM system to image cerebral blood vessels in living mice with an acquisition time about 4 min (Yao and Wang, 2013). The system’s lateral and axial resolutions were 57 μm and 38 μm, respectively, and the maximum imaging depth in the mouse brain and abdomen were 3.2 mm and 4 mm, respectively (Figure 2B). As for PACT, the determinants of its spatial resolution are generally considered from two aspects, one is the acoustic diffraction limit, and the other is the directionality of the transducer elements (Cox et al., 2012). Furthermore, there are full ring or linear transducer arrays with spatial resolutions ranging from tens of microns to sub-millimeter for small animal brain imaging (Lin et al., 2015; Li et al., 2016; Zhang et al., 2018). Li et al. employed the PACT system with ring-shaped ultrasonic transducer array (UTA) to observe cerebral cortical blood vessels (Li L. et al., 2017), which integrated high spatiotemporal resolution (125-μm in-plane resolution, 50 μs/frame data acquisition, and a 50-Hz frame rate) with deep penetration capabilities in a coronal view of the rat whole brain (11 mm) (Figure 2C). Besides, Li et al. utilized the PACT system with a linear-shaped UTA to observe the coronal plane of a mouse brain (Li L. et al., 2018), which provided a lateral resolution of 75 μm and a sectioning thickness of approximately 0.5 mm within the depth of focus, effectively covering the entire mouse brain. The frame rate of the imaging system is 10 Hz (Figure 2D). In conclusion, PA imaging methods with different system configurations can exert their advantages in different experimental scenarios, among which PAM is mainly applied to high spatial-resolution imaging of cortical regions, and the superiority of PACT is imaging depth, which can be well applied to brain imaging, but with relatively low spatial resolutions. Finally, the summary of PA imaging experimental setups are displayed in Table 2.

**Table 2:** Summary of PA imaging experimental setups.

	Imaging resolution	Imaging depth	Imaging speed	Laser type	Wavelength	Energy used	Typical applications
OR-PAM	The lateral and axial resolutions were 3 μm and 15 μm in clear media^*^.	0.7 mm (through an intact skull with the scalp removed)^*^.	About 15 s when imaged the region at the size of 5 × 10 mm^2*^.	Single wavelength lasers with 3-ns and 3-ps pulse width^*^.	532 nm^*^.	<20 mJ/cm^2^ (Li B. et al., 2023; Li D. et al., 2023).	Noninvasively imaging human microvasculature (Li B. et al., 2023; Li D. et al., 2023).
AR-PAM	The lateral and axial resolutions were 57 μm and 38 μm by imaging a 6 μm carbon fiber^**^.	3.2 mm (through the intact skin and skull of a mouse *in situ*)^**^.	Image acquisition time of whole brain was about 4 min^**^.	OPO laser with 8-ns pulse width^**^.	560–580 nm^**^.	~12.1 mJ/cm^2**^.	Used to study skin diseases (Fakhoury et al., 2024).
PACT with ring UTA	The spatial resolution was 125 μm^***^.	11 mm (in a coronal view of the rat whole brain)^***^.	A frame rate of 50 Hz^***^.	Single wavelength lasers with 5–9 ns (1,064 nm) and 12-ns (720 nm) pulse width; OPO laser with 6-ns pulse width (630 and 680 nm)^***^.	630, 680, 720, and 1,064 nm^***^.	~8 mJ/cm^2^ for 630, 680, and 720 nm; ~18 mJ/cm^2^ for 1,064 nm^***^.	Small animal whole body imaging^***^.
PACT with linear UTA	The lateral resolution was 75 μm^****^.	The whole mouse brain was covered (sectioning thickness is ~0.5 mm within the depth of focus)^****^.	A frame rate of 10 Hz^****^.	Single wavelength laser with 4–6 ns pulse width^****^.	1,064 nm^****^.	~64 mJ/cm^2****^.	Small animal deep brain imaging^****^.

^*,**,***,****^Annotated information comes from the references of Yao et al. (2015), Yao et al. (2013), Li et al. (2017), and Zhang et al. (2018), respectively. OR-PAM, optical-resolution photoacoustic microscopy; AR-PAM, acoustic-resolution photoacoustic microscopy; PACT, photoacoustic computed tomography; UTA, ultrasonic transducer array, ns, nanosecond; ps, picosecond; OPO, optical parametric oscillator.

### 3.2 Structure changes in AD observed with PA

#### 3.2.1 Aβ plaques

**Figure 3:**
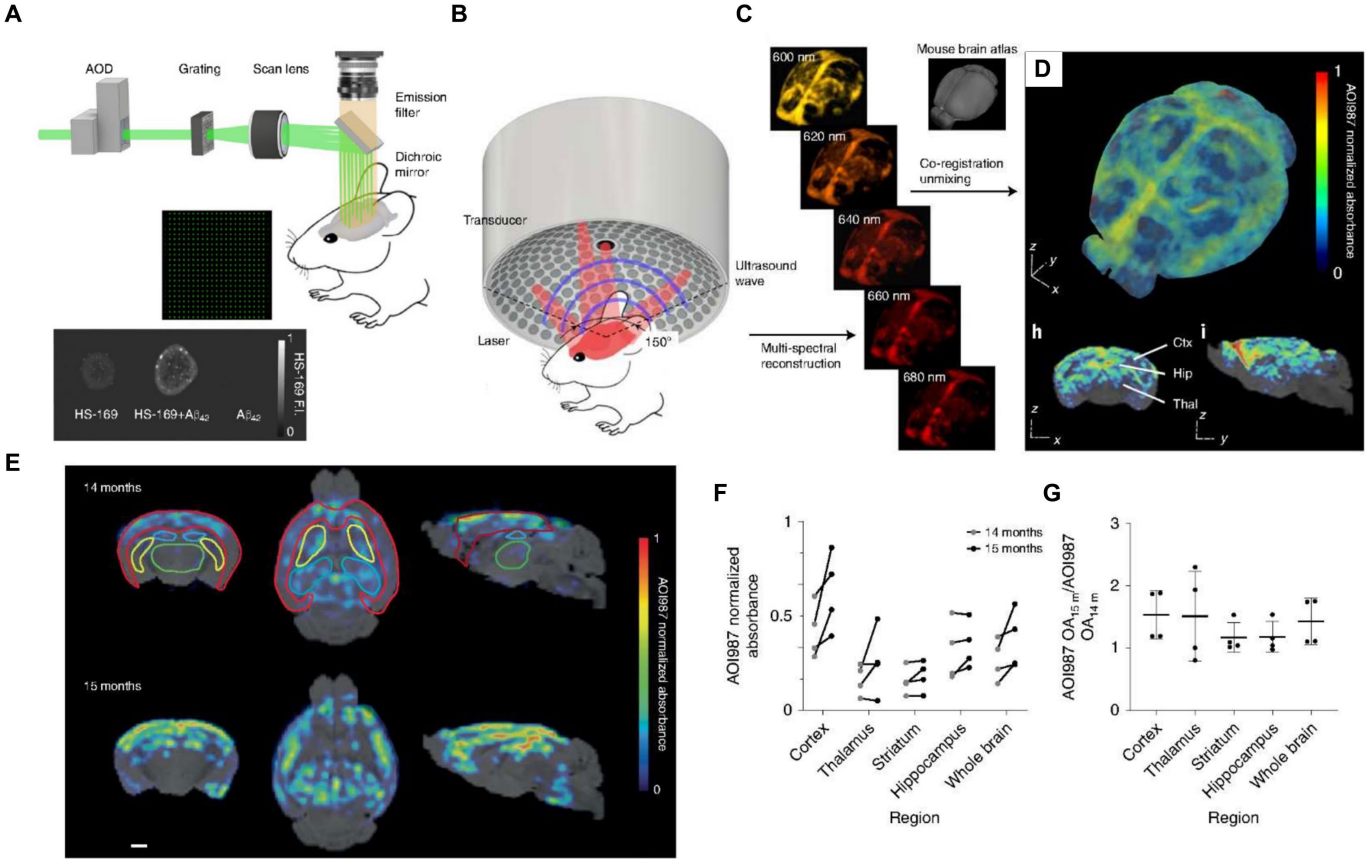
Fluorescence microscopy equipped with large-field multifocal illumination (LMI) fluorescence microscopy and panoramic volumetric multispectral optoacoustic tomography (vMSOT) for AD brain imaging studies. **(A)** Schematic diagram of LMI fluorescence microscopy system. AOD, acousto-optic deflector. **(B)** Schematic diagram of volumetric multispectral optoacoustic tomography (vMSOT) system. **(C)** The mouse brain was observed by the vMSOT system, and the test data was unmixed to reconstruct the images of different wavelengths. **(D)** 3D imaging of unmixed mouse brain showing the distribution of AOI987. Scalebar = 1 mm. **(E)** *In vivo* imaging of 14- and 15-month-old arcAβ mice performing by the vMSOT system shows the distribution of AOI987 and its co-localization with MRI images. The different colors of red, green, yellow, and blue are indicates cortex, thalamus, striatum, and hippocampus, respectively. Scalebar is 1 mm. **(F)** Quantitative assessment of AOI987 retention across various brain regions of the same mouse. **(G)** Statistics of the ratio of AOI987 signal intensity in different brain regions of 15-month-old mice to 14-month-old mice (n = 4). Reprinted with permission from Ni et al. (2022). Copyright 2022 Springer Nature.

Aβ plaque accumulation in the cerebral cortex and hippocampus is often considered the primary pathological hallmark of AD (de la Monte and Tong, 2014), leading monitoring Aβ plaque deposition to an essential procedure for the diagnosis and evaluation of AD. Ni et al. used the luminescent conjugated oligothiophene (HS-169) and the oxazine derivative (AOI987), fluorescent probes, which can target to Aβ plaque, to transcranially detect Aβ in the cortex of APP/PS1 mice. In this work, by combining the fluorescence microscopy with large-field multifocal illumination (LMI) with panoramic volumetric multispectral optoacoustic tomography (vMSOT), the arcAβ mice were imaged at 8-μm resolution for single plaque and at sub-150-μm resolution for the whole brain. These results are difficult to be acquired through conventional intravital microscopy (Ni et al., 2022). By combining the exceptional scanning capabilities of the optical deflector along with the uniform diffraction properties of the custom beam splitting grating, this approach efficiently employs a 21 × 21 micro-beam to conduct rapid scanning (Figure 3A). To verify the *in vitro* targeting performance of the probe, dispersions of HS-169 and Aβ_1-42_ fibrils were observed by LMI fluorescence microscopy. While possessing remarkable transcranial imaging capabilities and the ability to resolve single plaques, its scope is confined to observing cortical Aβ deposits. Therefore, it needs to implement the vMSOT system to further study non-invasive real-time whole-brain imaging With spatial and temporal resolution as well as penetration depth that surpasses conventional optical microscopy by a significant margin (Figure 3B; Razansky et al., 2009; Deán-Ben et al., 2016; Omar et al., 2019; Razansky et al., 2021). The vMSOT imaging progress includes multi-wavelength 3D image reconstruction, spectral separation of different tissue chromophores and exogenous administration of AOI987 (Figure 3C), and MRI-based co-registration mouse brain images. (Figure 3D). This method can not only distinguish Aβ deposition in different areas of the brain, but also identify brain protein deposition at different ages. For detail, the researchers initially assessed the system’s capacity to track alterations in Aβ within the brains of the same mice over time (Figure 3E). The age-related retention capacity of the AOI987 probe in different brain regions can be assessed by testing the same mice at 14 and 15 months of age, respectively, (Figure 3F). Specifically, at 15 months old, the unmixed AOI987 signal in mice showed a 30% increase in comparison to their state at 14 months old (Figure 3G), which is aligns with previous researches (Knobloch et al., 2007; Klohs et al., 2013).

#### 3.2.2 Neurovascular

**Figure 4:**
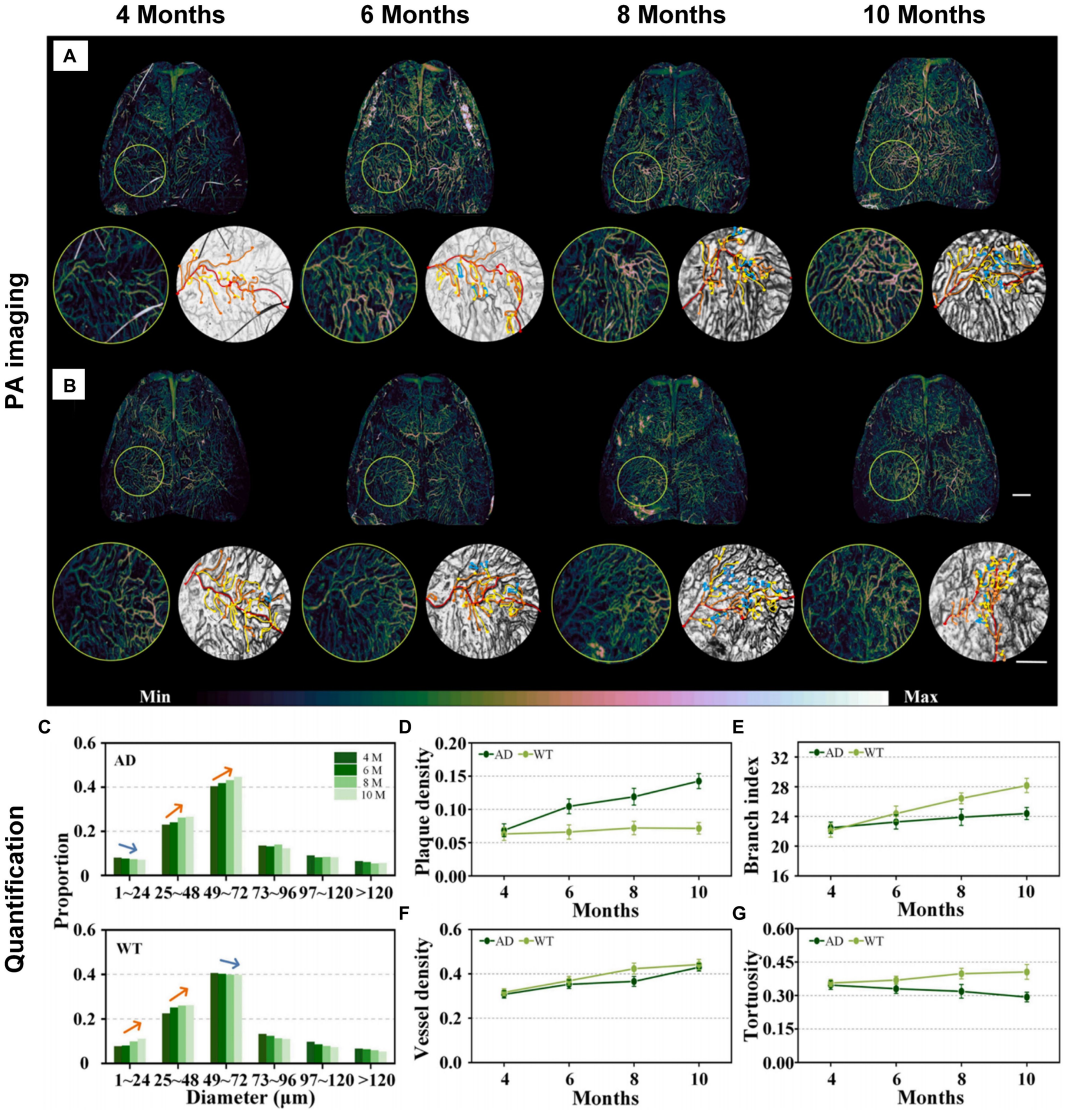
Imaging and measuring AD and WT mice at different time points (4, 6, 8, and 10 month). **(A,B)** Neurovascular imaging of the whole-brain meninges and cortex in AD and WT mice. Scalebar in **(B)** is 1 mm. Color bar in **A** and **B** represents the PA signal intensity. **(C)** Blood vessel diameter measurement analysis results in **A,B**. **(D–G)** Plaque density, vessel density, branching index and tortuosity in AD and WT mice (*n* = 3) of different ages. Reprinted with permission from Guo et al. (2023). Copyright 2023 Elsevier.

AD, being a neurodegenerative disease, manifests various characteristics across different stages. Liu et al. devised an arched-scanning PA microscopy (AS-PAM) with homogeneous-resolution, performing extensive imaging of the mouse cerebral cortex with a broad field of view (FOV) (Guo et al., 2023). In the pathology of AD, changes in neurovascular structure and function can be identified through electron microscopy due to proteins deposited on the cerebral cortex and blood vessels (Meyer et al., 2008). Nevertheless, the precise localization and patterns of neurovascular lesions in AD remain uncertain, especially *in vivo*. Recognizing the proven benefits of comprehensive imaging with consistent resolution for accuracy in authenticity and quantification (Xu et al., 2019), researchers use this approach to obtain more comprehensive and quantified outcomes compared to localized observations. For study on patterns of neurovascular alterations in the brain, The AS-PAM system was employed for neurovascular imaging and quantitative analysis in mice at different stages of AD and wild-type (WT) mice (APP/PS1 genetically engineered mouse strain). Figures 4A,B present neurovascular imaging of the whole-brain meninges and cortex in AD and WT littermate mice at 4, 6, 8, and 10 months of age, respectively. The quantized results show that AD mice have lower microvascular production than WT mice (Figure 4C). Apparently, Aβ plaque deposition in the brains of AD mice increases with age, while the deposition level in WT mice remained stable (Figure 4D). More importantly, the vascular branching index (a neovascularization parameter (Zudaire et al., 2011)) of AD mice has a slight increase, while WT mice showed an obvious upward trend (Figure 4E). However, cerebral blood vessel density in AD mice and WT mice showed the same trend, increasing with age (Figure 4F). Finally, the blood vessel tortuosity decreased in AD mice but steadily increased in WT mice (Figure 4G). The findings from this detection align with earlier studies through *in vitro* electron microscopy, suggesting that Aβ plaques adhere to and accumulates on the walls of meningeal blood vessels and cortical spaces in AD mice. This presence affects regular angiogenesis, potentially leading to cell apoptosis and morphological alterations in blood vessels (Boche et al., 2008; Nasiriavanaki et al., 2014). Besides, a distinct result uncovers that despite comparable blood vessel density, AD mice exhibit lower proportions of neovascularization and lower branch index compared to WT mice. This work analyzes changes in the entire cerebrospinal fluid and cortical vascular system during the progression of AD *in vivo*, highlighting vascular features as indicative biomarkers of AD progression. Conclusively, this research has a contribution to monitoring and evaluating the progression of brain diseases by utilizing homogeneous-resolution and a wide FOV to capture images of the entire brain.

### 3.3 Functional (microenvironmental) changes in AD obtained with PA

#### 3.3.1 Blood oxygenation

Various endogenous probes can be used to study not only tissue structural information, but also the multi-functional information. Hemoglobin is usually selected as the absorber because of its robust absorption in visible light, where the absorption peak of oxyhemoglobin (HbO_2_) is at 850 nm, while the absorption peak of deoxyhemoglobin (HbR) is around 750 nm (Beard, 2011; Xia J et al., 2014). Based on this property, PA imaging can be exploited to visualize blood vessels and measure blood oxygen saturation, calculated by Eq. (1):


(1) $$sO_{2}=\frac{C_{HbO_{2}}}{C_{HbO_{2}}+C_{HbR}}\times 100\%$$

where $C_{HbO_{2}}$ and $C_{HbR}$ represent the concentration of $HbO_{2}$ and HbR seperately. In PA imaging, the values $C_{HbO_{2}}$ and $C_{HbR}$ are obtained, respectively, through multispectral imaging, then sO_2_ measurement can be achieved (Li M et al., 2018), which, typically, falls within the range of 0.95 and 1 in arteries and from 0.66 to 0.8 in veins (Harrop, 1919). A linear unmixing algorithm is commonly used in PA imaging to quantify the blood vessel sO_2_ based on the acquired PA signals of two wavelengths, calculated by Eq. (2) (Liu and Wang, 2022):


(2) $$sO_{2}=\frac{\varepsilon_{2}^{de}-\rho \varepsilon_{1}^{de}}{\left(\varepsilon_{2}^{de}-\varepsilon_{2}^{oxy}\right)-\rho \left(\varepsilon_{1}^{de}-\varepsilon_{1}^{oxy}\right)}$$

where ρ is the ratio between the normalized PA amplitudes of wavelength $\lambda_{2}$ and that of $\lambda_{1}$ , $\varepsilon_{i}^{oxy}$ , $\varepsilon_{i}^{de}$ are the molar extinction coefficients of HbO_2_ and HbR at the wavelength $\lambda_{i}$ .

**Figure 5:**
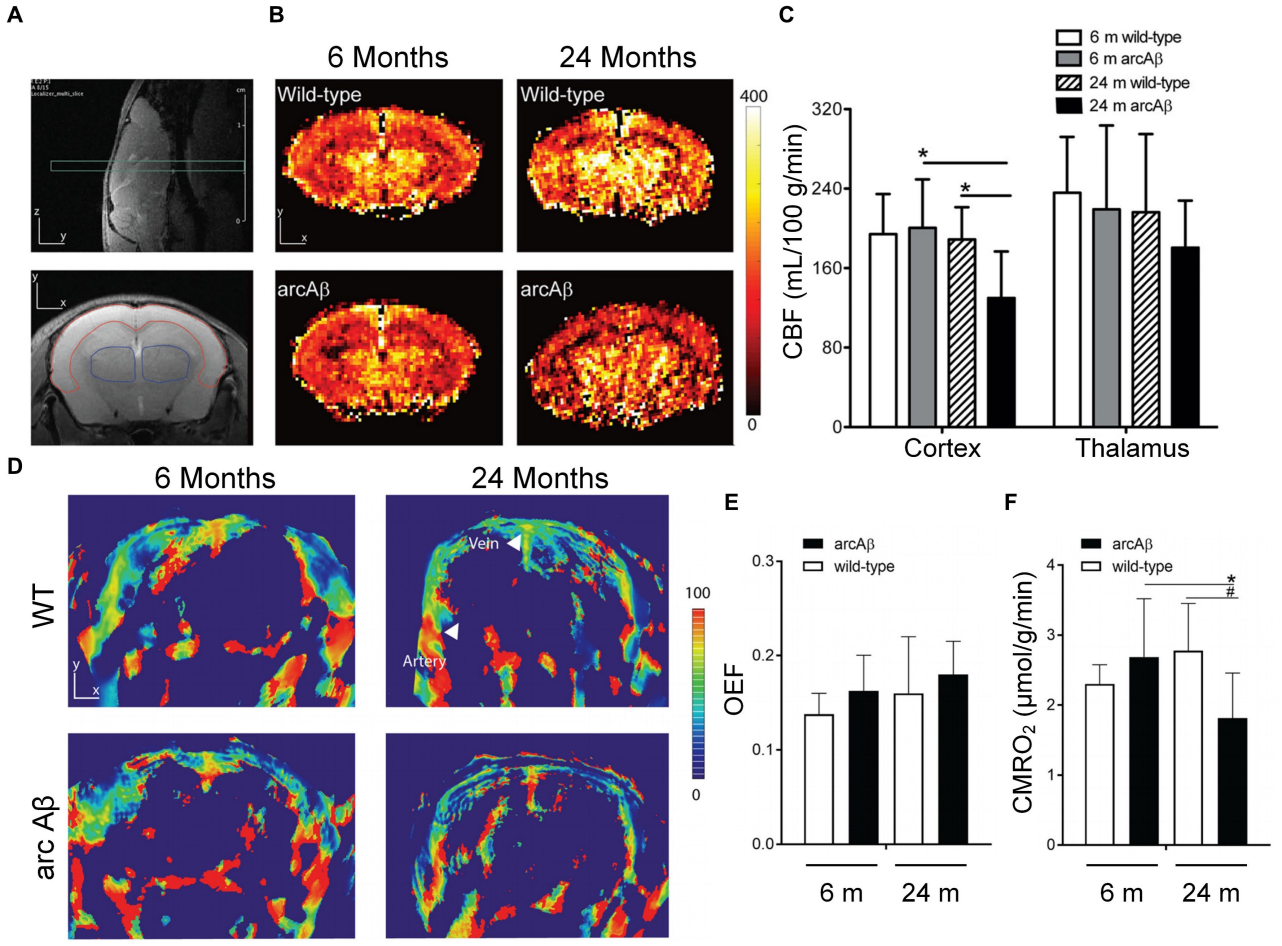
Cerebral perfusion MRI and PACT were performed to detect cerebral blood flow (CBF), oxygen extraction fraction (OEF), and cerebral metabolic rate of oxygen (CMRO_2_) in young and old WT mice and arcAβ mice. **(A)** Coronal imaging of mouse brain using T_2_-weighted perfusion MRI. The area surrounded by red line is the cerebral cortex, and the thalamus is surrounded by the blue one. **(B)** Coronal CBF images of the brains of young and old WT and arcAβ littermate mice. **(C)** CBF statistics of the cortex and thalamus in **B**. **(D)** Coronal blood oxygen saturation (SO_2_) images of the brains of young and aged WT and arcAβ littermate mice. **(E)** Quantitative results of OEF and **(F)** CMRO_2_ based on the information in **D**. Reprinted with permission from Ni et al. (2018). Copyright 2018 Elsevier.

However, the balance of oxygen transport and consumption may be affected by certain diseases, making sO_2_ as a potential indicator for disease early screening. For instance, studies of neurological diseases can be conducted by quantifying sO_2_ and by measuring cerebral blood flow (CBF) using MRI, which can manifest brain functional physiology (Clarke and Sokoloff, 1999). In some cases, the behavior of the nervous system can also be monitored by testing brain oxygen extraction fraction (OEF) via Eq. (3) (Lin et al., 2023):


(3) $$OEF=\frac{S_{a}O_{2}-S_{v}O_{2}}{S_{a}O_{2}}$$

where s_a_O_2_ is arterial oxygen saturation, while s_v_O_2_ is the venous oxygen saturation. In addition, cerebral oxygen metabolic rate (CMRO_2_) can also be calculated and visualized from oxygen gradient in arteries and veins, according to Fick’s principle, with Eq. (4) (Biondetti et al., 2023):


(4) $$CMRO_{2}=C\mathrm{BF}*\left(S_{a}O_{2}-S_{v}O_{2}\right)*C_{a}$$

where *C_a_*, is a constant indicating the maximum capacity for unit volume of blood to deliver oxygen. Reported by Ni et al., the decreased cortical vascularity and reduced CMRO_2_ were found by combined PACT and perfusion MRI in transgenic arcAβ mouse models (Ni et al., 2018). In this work, by employing five wavelengths (715, 730, 760, 800, and 850 nm), the cortical tissue oxygenation can be estimate by evaluating cortical vessel oxygenation, s_a_O_2_ of the middle cerebral arterial, and s_v_O_2_ of the superior sagittal sinus (Ni et al., 2018). Other work shows that no statistical differences are observed in OEF or CMRO_2_ between young and aged WT mice by imaging and quantitative analysis, while CMRO_2_ was obviously reduced in aged arcAβ mice than in young ones. The accumulation of Amyloid plaque and amyloid angiopathy can be found in arcAβ mice and old wild mice, but not in young wild mice, which indicates that the reduction of CMRO_2_ in arcAβ is not only caused by age factors, but is closely related to protein pathology shown in Figure 5 and Ni et al. (2018).

#### 3.3.2 Meningeal lymphatics

**Figure 6:**
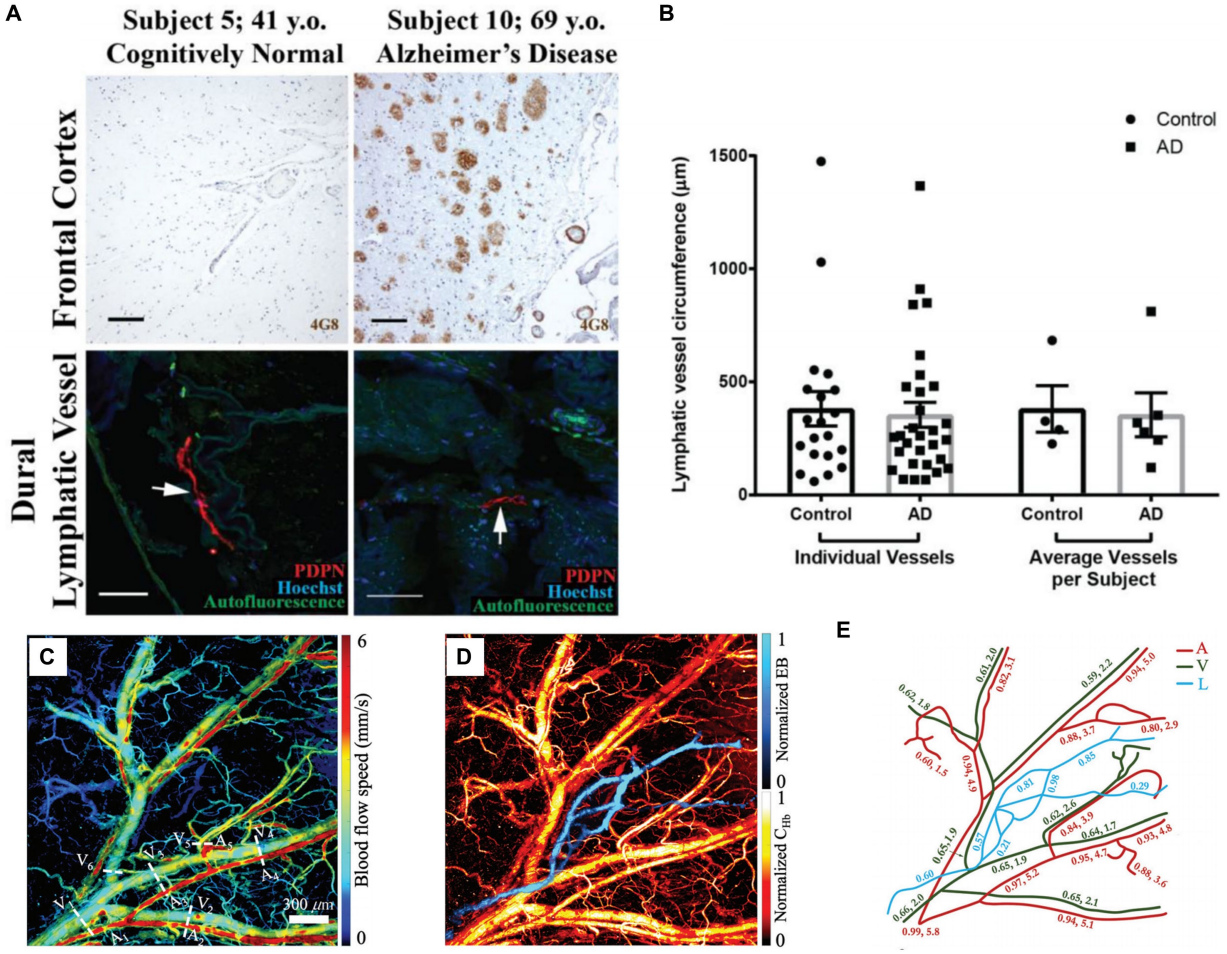
**(A)** Aβ plaque deposition in frontal cortex and leptomeningeal vessels in control and AD groups. **(B)** Lymphatic vessel circumference measurements. Reprinted with permission from Goodman et al. (2018). Copyright 2018 Elsevier. **(C)** Blood flow speed in the vessels of the mouse ear. Five arteries (A) and six veins (V) are marked in the image. **(D)** PAM images of blood vessels and lymphatic vessels (L) in mouse ears. **(E)** Changes of SO_2_, relative lymph concentration and blood flow speed in different parts of mouse ear. Reprinted with permission from Liu C. et al. (2021). Copyright 2021 SPIE.

Researches have highlighted the significance of meningeal lymphatic vessels as a pathway crucial for eliminating waste deposited in the central nervous system (Aspelund et al., 2015; Louveau et al., 2015), such as draining macromolecules from the central nervous system to cervical lymph nodes, which plays an important role in maintaining brain homeostasis. Da Mesquita et al. found that brain dysfunction occurs when the function of meningeal lymphatic vessels is impaired (Da Mesquita et al., 2018). Goodman et al. used confocal microscopy to observe the structure of two types of human brain lymphatic vessels and verified through experimental results that lymphatic vessels play an important role in clearing Aβ plaques (Goodman et al., 2018). As shown in Figure 6A, 6E10 and 6G Aβ antibody clones were used to label sequential cortical and superior sagittal sinus. The former one can only bind fibrillar Aβ, while the later one can bind both fibrillar and prefibrillar oligomeric. The frontal cortex of the control group did not show immunofluorescence of Aβ plaques, but dense Aβ immunoreactivity were observed in brain sections of AD patients. In addition, in order to verify whether there are differences in the structure of meningeal lymphatic vessels between normal and AD subjects, the circumference of lymphatic vessels was measured. The results showed that there was no significant difference in the circumference of lymphatic vessels between the two groups (see Figure 6B).

Besides fluorescence imaging (FLI), PA imaging can also demonstrate the research on lymphatic vessels *in vivo*. Liu et al. used a five-wavelength OR-PAM to perform simultaneous imaging the blood and lymphatic vessels of the mouse ear yielded rich structural and functional information (see Figures 6C–E) (Liu C. et al., 2021). The lymphatic vessels were labeled by the Evens Blue, which is sensitive to light with a wavelength of 620/640 nm. By taking images at different time, the lymphatic clearance process can be monitored through PA. Although there are currently no studies on meningeal lymphatics using PA technology, it can be speculated from the above studies that PA imaging has the potential to study meningeal lymphatics and cerebral vessels together, providing more structural and functional information for brain research.

### 3.4 Probe-enhanced PA imaging of AD

AD is mainly judged by the deposition of Aβ plaque. Its main accumulation locations include leptomeningeal arteries, cortical arteries and veins in brain (Weller et al., 1998). Cerebral amyloid angiopathy (CAA) is widespread among most individuals with AD (Liu Y. et al., 2017). Considering the possibility that Aβ deposits can develop concurrently or independently in the walls of blood vessels and brain parenchyma (Ma et al., 2020), developing an exact strategy is essential to accurately map these anomalies, enabling the effective identification of CAA in live subjects. As highly scattering and absorbing media, the gray and white matter of the brain pose a huge obstacle to deep brain FLI, severely limiting its application in plaque localization (Ma et al., 2020).

**Figure 7:**
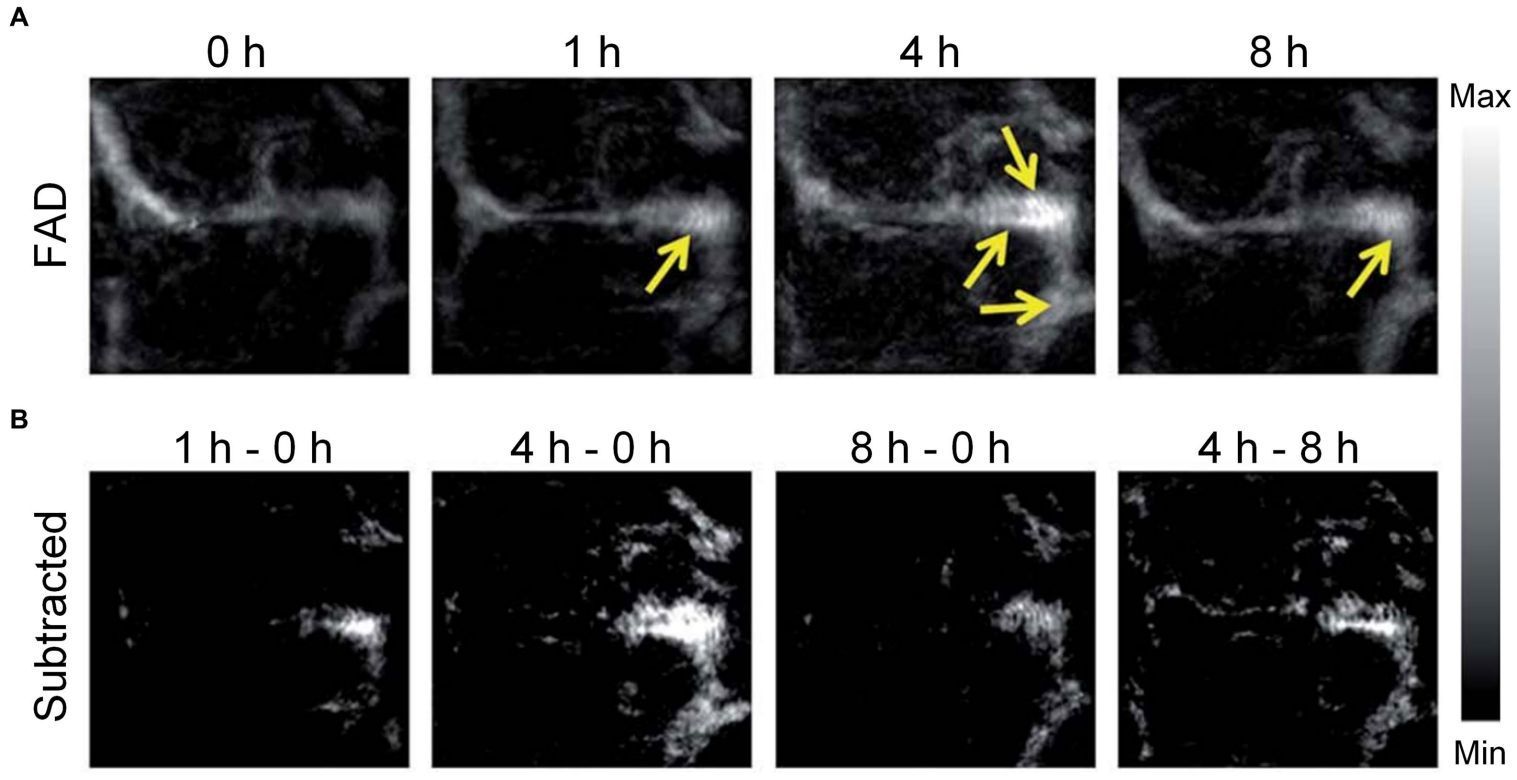
PA imaging was performed on the brain plaques in mice brains. **(A)** PA imaging recorded maximum amplitude projection images captured in Tg mice post-injection of contrast agents at different time points; brain plaques within the mouse blood vessels are indicated by yellow arrows. FAD, familial Alzheimer’s disease. **(B)** Enhancement of PA signals in the brains of Tg mice. Reprinted with permission from Liu Y. et al. (2017). Copyright 2017 Royal Society of Chemistry.

In contrast to FLI, PA imaging has the capability to acoustically overcome intense optical scattering while maintaining considerable penetration depth and spatial resolution. It is achieved through the utilization of PA contrast agents or micro robot, significantly improving the efficiency and accuracy of PA imaging (Yin et al., 2018). In particular, Zhang et al. utilized the sensitive fluorescent and PA dual-modal H_2_S probes based on nitrobenzoxadiazole (NBD) amine to explore H_2_S biology and diagnose H_2_S-related diseases (Zhang et al., 2021). On the other hand, Yu et al. designed paramagnetic nanoparticles (SiO_2_ coated magnetite particles) with adjustable area concentration, which can enhance the contrast of PA imaging by driving the degree of nanoparticle aggregation (Yu et al., 2019). Futhermore, Li et al. used 3D printing technology with nanoscale resolution to fabricate micro-rocket robots (SU-8, a kind of photoresists) with high driving capability to achieve high-resolution PA vascular imaging via an OR-PAM system (Li D. et al., 2020). Liu et al. pioneered the evaluation of a unique PA imaging probe (an NIR ultrahigh absorbing croconium dye for amyloid) engineered specifically for imaging Aβ plaques in a transgenic (Tg) mouse model, eliminating the need for antibody labeling (Liu Y. et al., 2017). Figure 7A displays the PA imaging of the mice’s brains at different time points after injecting of the contrast agent. The PA images reveal a notable increase in the PA signal from the organic dye in the cerebral blood vessels of the Tg group, demonstrating a considerable buildup of Aβ fibers. Figure 7B shows the differential PA images, obtained by subtracting the initial image acquired immediately after drug injection from the subsequent PA images captured at different time points. It is very obvious that the PA imaging contrast within the sagittal sinus is greatly improved when comparing the PA images of the former 4 h to these of later 4 h after injection. The phenomenon might be associated with the generation of Aβ plaques and the dye gradually accumulated in the cerebral blood vessels. After 8 h, there was a apparent decrease of the PA signals in the brain vessels of the Tg group, which may be caused by that small molecule dyes are metabolized from the brain. These findings confirm the effectiveness of PA imaging in identifying brain plaques on cortical vessels. The applications of PA imaging in diagnosing AD were summarized in Table 3, which benefits to more directly understand.

**Table 3:** Summary of PA imaging researches on AD.

Methods	Wavelength used (nm)	Advantages	Shortcomings
Fluorescence microscopy equipped with LMI and vMSOT (Ni et al., 2022).	600, 620, 640, 660, and 680 nm	Transcranial detection; Distinguishing Aβ deposition in different brain regions and at different ages.	Limited axial resolution; Inability to visualize small blood vessels; lack of clear anatomical brain landmarks in the vMSOT images.
Arched-scanning AS-PAM with homogeneous-resolution (Guo et al., 2023).	532 nm	Ultrawide FOV to cover the entire mouse cerebral cortex; Precise brain neurovascular visualization and quantification.	Hair and scalp removal.
Combining PACT and perfusion MRI (Ni et al., 2018).	715, 730, 760, 800, and 850 nm	Non-invasive detection of physiological changes in vascular and tissue oxygenation.	Cannot assess oxygenation in the microvasculature; Cannot provide absolute quantification of SO_2_.
Multifunctional probe be used in PAT/PET/FLI (Liu Y. et al., 2017).	800 nm	Without antibody linkage; Multifunctional probe for imaging.	Probe dependency.

**Figure 8:**
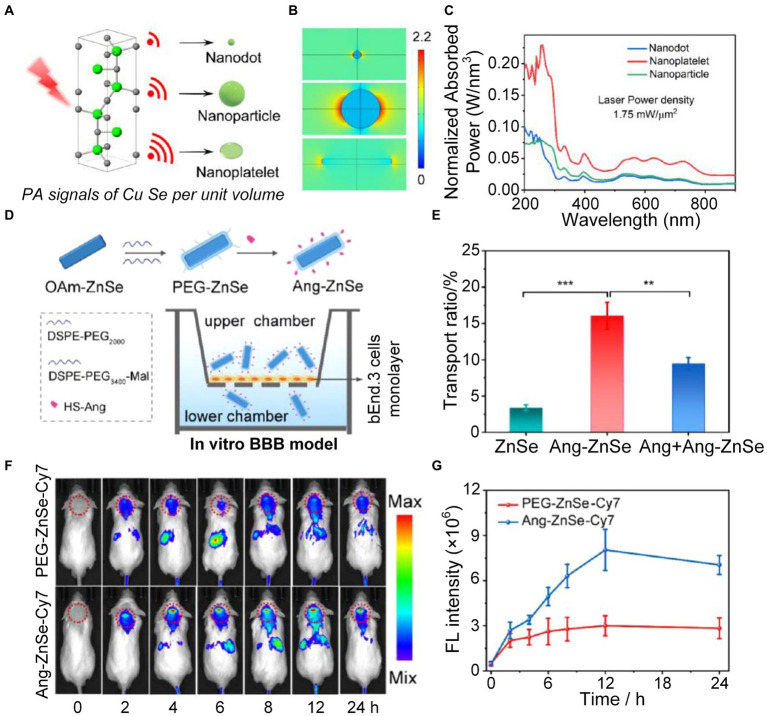
Research on ZnSe nanoplatelets monitoring the level of Cu^2+^ in mice brains. **(A)** Schematic diagram of PA signal intensity generated by CuSe nanoprobes with different shapes. **(B)** Electric field distribution of CuSe nanoprobes in different shapes under light excitation. **(C)** Absorption spectra of three different shapes of CuSe under the same laser power density. **(D)** Schematic diagram of *in vitro* experiments displaying nanoprobes crossing the blood–brain barrier (BBB). **(E)** Evaluation of the ability of ZnSe nanoprobes with three different conditions to cross the BBB. **(F)** *In vivo* fluorescence imaging (FLI) at 0, 2, 4, 6, 8, 12, and 24 h after injection of PEG−ZnSe−Cy7 or Ang − ZnSe−Cy7 nanoplatelets. Color bar represents the fluorescence signal intensity. **(G)** Statistics of fluorescence intensity changes over time in the red dotted circle in **F**. Reprinted with permission from Han et al. (2022). Copyright 2022 American Chemical Society.

The onset of AD may also be attributed to a variety of causative factors, including excitotoxicity, dysregulation of transition metals, decreased levels of natural antioxidants, and neuroinflammation, which can cause neuronal injury (Bush, 2003; Papadia et al., 2008; Park et al., 2018; Ma et al., 2020). Excessive buildup of redox-active metal ions, particularly copper (Cu^2+^), results in heightened oxidative stress within the brain (Multhaup et al., 1996; Barnham et al., 2004; Pedersen et al., 2016; Reybier et al., 2016). This condition caused harm to cellular components and impact on signal pathways linked to degenerative neurological disease (Block et al., 2007; Liu W. et al., 2017; Kausar et al., 2018). To better understand the dysregulation of copper homeostasis in AD, it’s vital to use non-invasive imaging methods to continually track the real-time changes in copper dynamics within the brain. Jiang et al., using finite element analysis (FEA) (Figures 8A–C) and related experiments, confirmed that the near-infrared absorption and PA pressure of copper selenide (CuSe) nano-platelets significantly increased compared with nanodots and nanoparticles (Han et al., 2022). This discovery promoted the progress of zinc selenide (ZnSe) nanoplatelet probes, enhancing PA imaging to detect *in situ* Cu^2+^ exchange within the brains of AD mice in real-time. In order to enhance the transit of nano-platelets across the BBB, the Angiopep-2 (Ang) peptide, which serves as a ligand specifically targeting the overexpressed lipoprotein receptor-associated protein 1 (LRP1) on the BBB, was intricately modified on the surface of the nano-platelets (Tang et al., 2019). The synthetic nanoplatelet probes efficiently traversed the BBB and promptly interacted with Cu^2+^ in brain, enhancing the ability of PA imaging to detect the level of copper ion in the brain (Figures 8D–G).

## 4 Treatment based on light and PA methods

### 4.1 PA cavitation therapy with metal–organic frameworks

**Figure 9:**
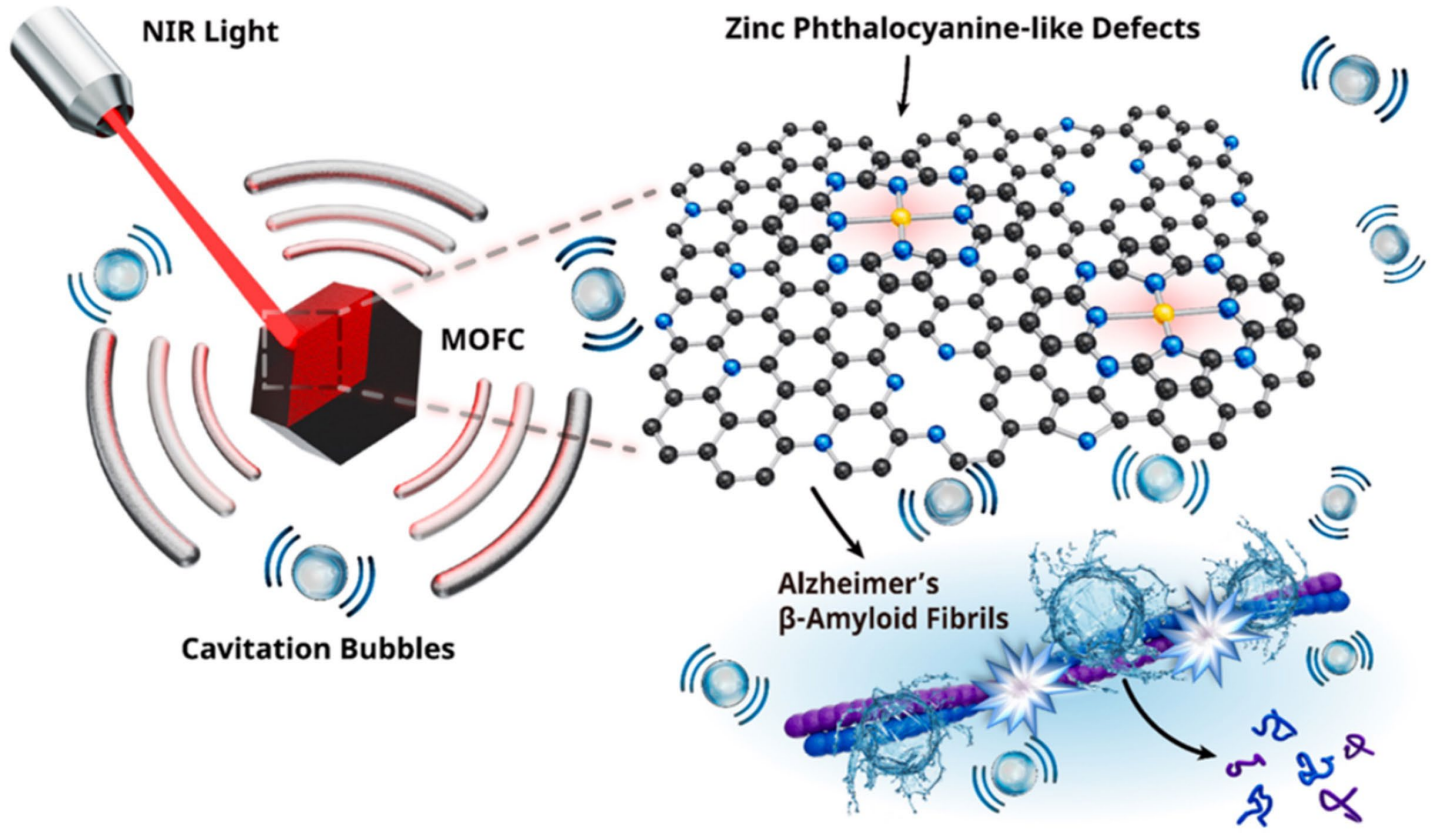
Schematic diagram of MOFC nanoparticles generating cavitation bubbles to degrade the Aβ fibril structure under near-infrared laser excitation. Reprinted with permission from Jang et al. (2022). Copyright 2022 American Chemical Society.

Given the neurotoxic effects of aggregation, preventing abnormal Aβ aggregation is considered a key strategy for the treatment of AD (Panza et al., 2019). One advantage for PA therapy is that the near-infrared light penetrating tissue can generate sound waves through PA effects, which induces cavitation bubbles in the body without the need to inject encapsulated microbubbles (Wang et al., 2020, 2021; Liu et al., 2022; Mi et al., 2023). PA cavitation can facilitate the use of intravenically encapsulated microvesicles [such as intravascular recalculation (Liu L. et al., 2019) and transient BBB opening (Jang et al., 2022)] in clinical applications, including the eradication of tumor lesions and the application of rapid sonic flow resulting from bubble rupture (Liu L. et al., 2019; Wang et al., 2020). Unlike intravenous microbubble injections, PA treatment can produce local and concentrated cavitation bubbles in specific body areas. This is due to the emission of photoconductive sound waves by PA agents, which only occur close to their structures. In addition, the PA mode has the advantage of potentially eliminating risks [e.g., hemolysis (Krasovitski et al., 2011), necrosis (Kim et al., 2019)] associated with external US stimuli used to activate encapsulated microvesicles. Jiang et al. revealed a new capability of metal–organic frame-derived carbon (MOFC) nanoparticles, demonstrating the ability of nanoparticles to trigger powerful PA emptiness upon near-infrared absorption, effectively destroying Aβ aggregates in AD (Figure 9) (Jang et al., 2022).

**Figure 10:**
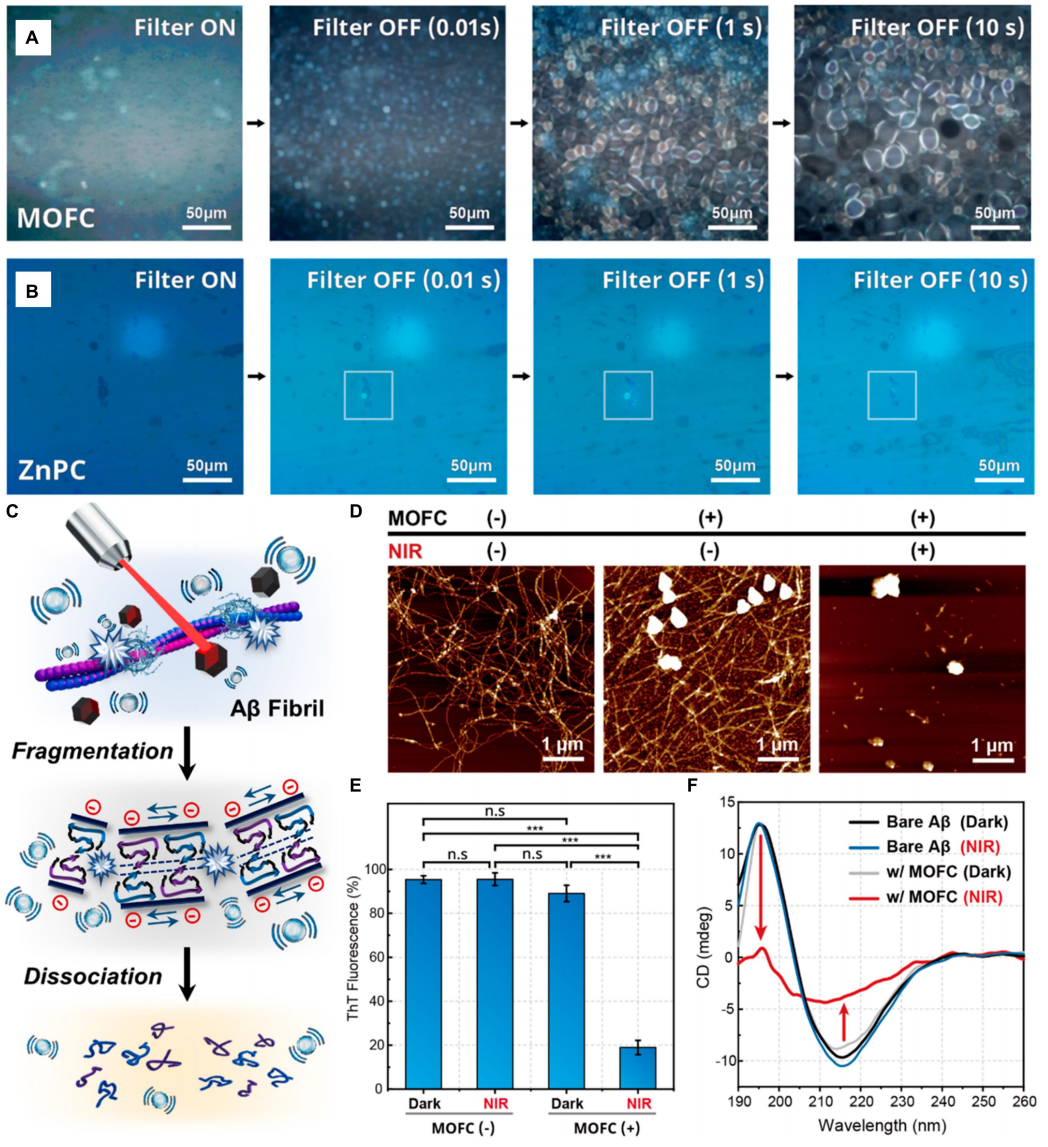
The *in vitro* cavitation experiment of MOFC and ZnPC, and the evaluation of MOFC’s ability to degrade Aβ fibril structure. Pictures of cavitation bubbles generated by **(A)** MOFC and **(B)** ZnPC nanoparticles under NIR laser excitation. **(C)** Schematic diagram of the structural dissociation of Aβ aggregates under the action of PA cavitation bubbles. **(D)** atomic force microscope (AFM), **(E)** thioflavin T (ThT) assay and **(F)** circular dichroism (CD) spectra were used to detect the situation of Aβ fibrils co-incubated with MOFC under 808 nm laser irradiation for 2 h, and compared with the results without irradiation. The nanoparticle concentration and laser power density are, respectively, 100 μg mL^−1^ and 1 W cm^−2^. Reprinted with permission from Jang et al. (2022). Copyright 2022 American Chemical Society.

The *in vitro* cavitation degradation experiment of Aβ fibrils shows that MOFC has better cavitation bubble generation performance compared with ZnPC (see Figures 10A,B). Under the excitation of near-infrared laser, the cavitation bubbles generated by MOFC nanoparticles can precisely affect the structure of Aβ fibrils, transforming them from a thermodynamically stable structure to a structure that is non-toxic to the brain (see Figure 10C). Microscopic and spectroscopic analyses reveal that PA cavitation disrupts the elongated formations of Aβ aggregates, fragmenting them into spherical structures. Additionally, it markedly diminishes the prevalence of β-sheet secondary structures within these aggregates (see Figures 10D–F). The results of this work indicate that the PA cavitation effect of MOFC nanoparticles induced by near-infrared laser can effectively destroy the structure of AB fibril aggregates and break them into small fragments, thereby reducing its toxicity to the brain. In addition, the PA cavitation disruption of Aβ aggregates does not consume the oxygen molecules dissolving in water or free charge carriers. The application of the cavitation effect excited by NIR light to non-invasively degrade Aβ aggregates that are toxic to the brain can provide broad prospects for the treatment of AD (Jang et al., 2022).

**Figure 11:**
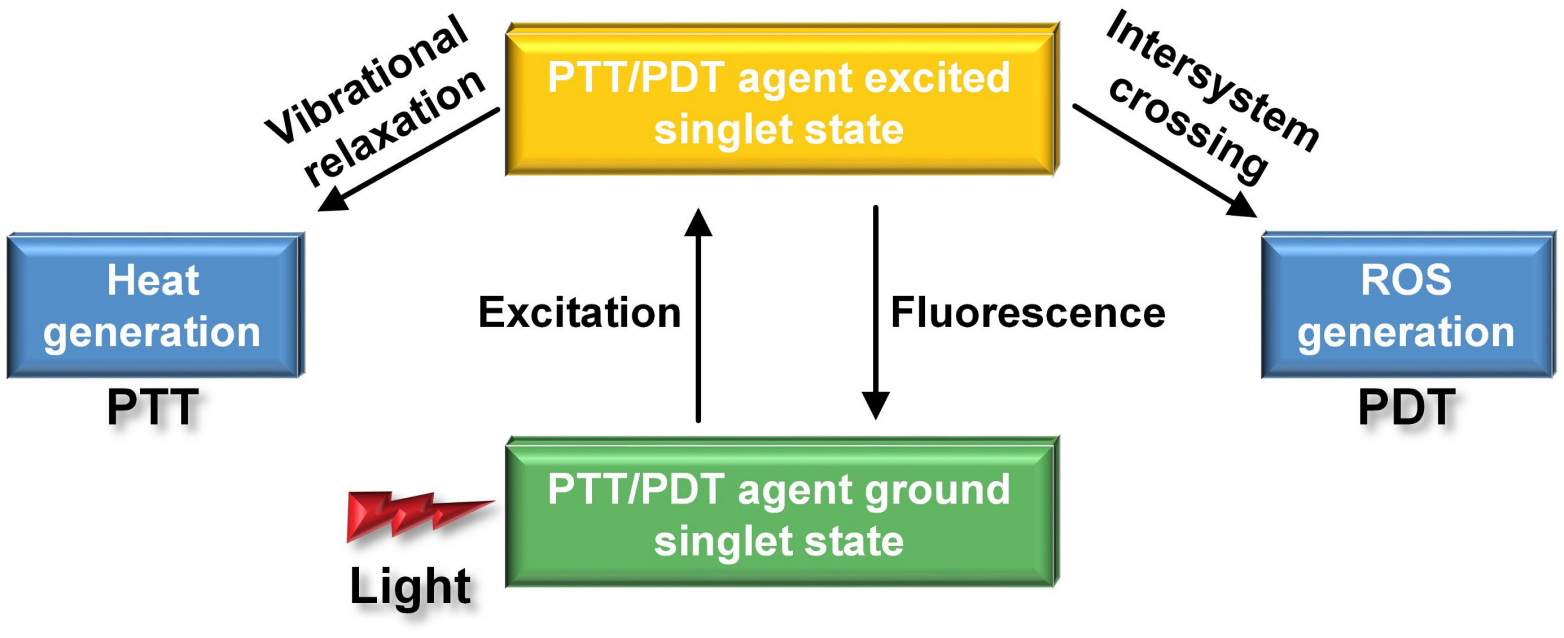
Different nanoparticles produce different effects under the action of laser. The PDT agent can produce ROS with strong oxidation effect, while the PPT agent generates heat.

Despite numerous clinical efforts to translate promising results from animal models of AD into viable treatments, including the use of inhibitors such as peptides, peptide mimetics (Goyal et al., 2017), small organic compounds (Frydman Marom et al., 2009), nanoparticles (Han et al., 2017), and Aβ-specific antibodies (Bu, 2009) to hinder Aβ aggregation and disassemble Aβ fibrils, there is still a lack of an approved and effective medicine for treating AD. There is an urgent need to explore novel therapeutic drugs or approaches to improve treatment outcomes for clinically silent AD. Phototherapy has emerged as an innovative treatment for cancer, thanks to its operational flexibility, noninvasive nature, and high spatiotemporal resolution, enhancing therapeutic effectiveness while minimizing systemic toxicity (Gao et al., 2020, 2021; Li X et al., 2020; Yang et al., 2020). Outstandingly, phototherapies have shown significant promise in the field of AD treatment (Hamblin, 2019). Two primary methods, known as PDT and PTT, are employed, the former relies on photosensitizer (PS), while the latter relies on photothermal transfer agents (PTAs). When PS interact with light, it initiates photochemical reactions, resulting in the generation of either reactive oxygen species (ROS). As for PTAs, it induces thermal effects when irradiating by light. Both ROS and thermal effects can destroy Aβ aggregates and reduce its cytotoxicity (Figure 11).

### 4.2 Photodynamic therapy

PDT utilizes non-toxic photosensitive substances exposed to specific wavelengths of light to induce phototoxicity in particular cancer cells or diseased cells to achieve therapeutic effects that have been demonstrated to kill microbial cells, including bacteria, fungi, and viruses. Singlet oxygen (^1^O_2_) is known for its powerful oxidation properties (Chilakamarthi and Giribabu, 2017; Kwiatkowski et al., 2018). The ROS produced in PDT have the capacity to oxidize amino acid residues, consequently inhibiting the assembly of AB monomers to form AB aggregates.

Since the 21st century, benefit from to the rapid development of nanotechnology, the technology for synthesizing upconversion nanoparticles (UCNPs) has become increasingly stable. UCNPs can absorb multiple (two or more) long-wavelength photons and release one short-wavelength photon. This characteristic makes them widely used in biofluorescence imaging (Liu et al., 2011), drug delivery (Starsich et al., 2017), photodynamic/photothermal therapy (Xia L. et al., 2014; Lucky et al., 2015), etc. Their exceptional optical properties make them highly versatile in the use of imaging techniques to diagnose tumors and various diseases.

**Figure 12:**
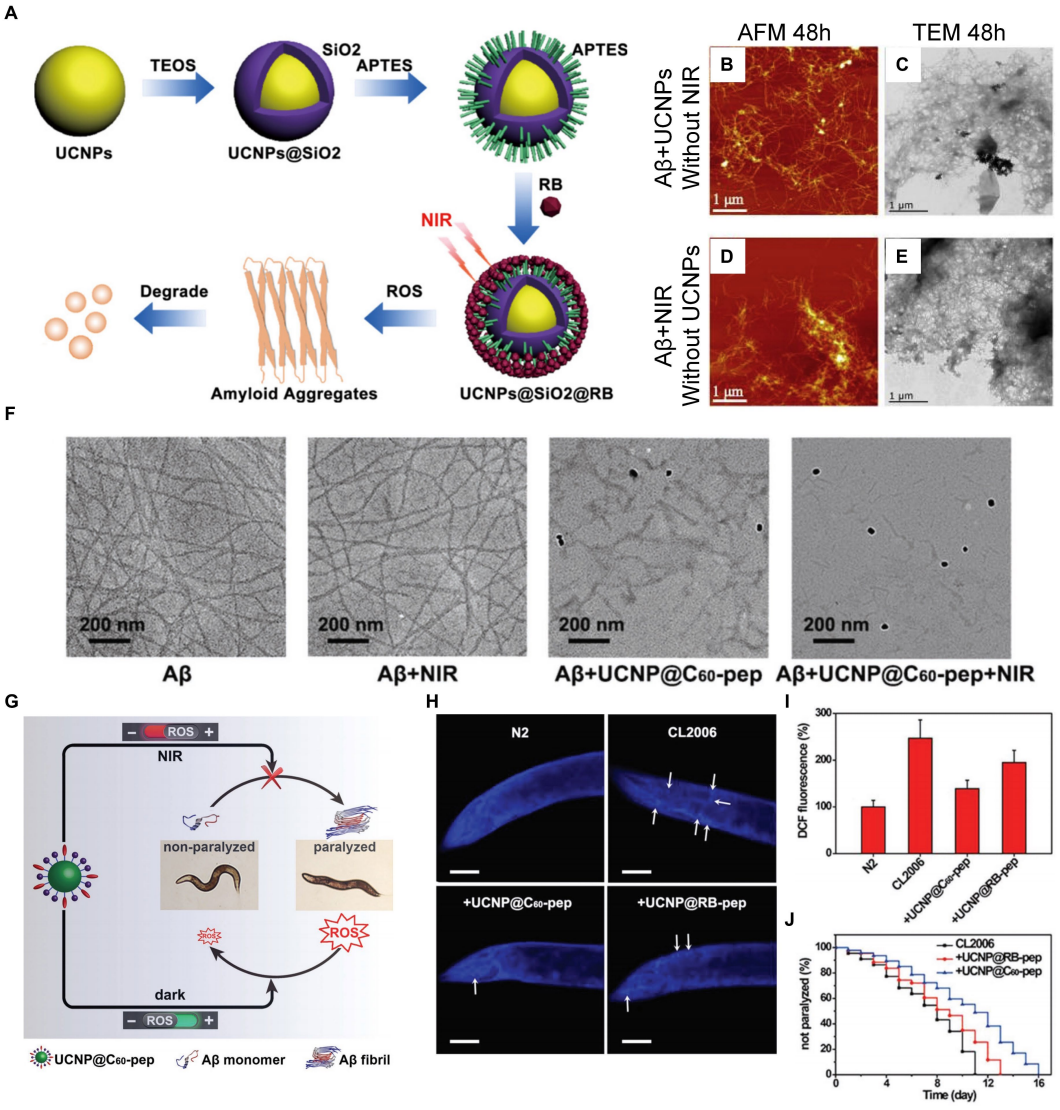
Researches on PDT of AD based on UCNPs. **(A)** The synthesis process of NaYF_4_:Yb/Er@SiO_2_@RB and the schematic diagram of the production of ROS which has a destructive effect on the structure of Aβ fibrils. **(B)** AFM and **(C)** transmission electron microscope (TEM) images of Aβ aggregates after incubation with NaYF_4_:Yb/Er@SiO_2_@RB for 48 h without 980 nm NIR light irradiation. **(D)** AFM and **(E)** TEM images of Aβ aggregates without co-incubation with NaYF_4_:Yb/Er@SiO_2_@RB under 980 nm NIR light irradiation. Reprinted with permission from Zhang et al. (2019). Copyright 2019 Elsevier. **(F)** TEM images depicting Aβ after various indicated treatments. **(G)** UCNP@C_60_-pep can generate ROS under NIR irradiation to destroy the structure of Aβ aggregates, thereby reducing its toxicity to CL2006. In the absence of NIR light, UCNP@C_60_-pep can eliminate excessive ROS and ensure the homeostasis of intracellular redox levels. **(H)** Thioflavin S (ThS) fluorescence staining images of CL2006 strains incubated with UCNP@C_60_-pep and UCNP@RB-pep for 6 days, respectively. The N2, a kind of wild-type strain, was set as control group. Scalebar is 40 μm. **(I)** Using dichlorofluorescin (DCF) fluorescence imaging to detect ROS levels of each strain on the 6th day. **(J)** Survival of CL2006 co-incubated with UCNP@C_60_-pep and UCNP@RB-pep. (132). Reprinted with permission from Du et al. (2018). Copyright 2018 Wiley-VCH.

In recent researches, UCNPs have shown significant development in advancing AD treatment. UCNPs, having the potential of photosensitization induced by NIR light to inhibit Aβ deposition, were designed and developed by Kuk et al. (2017). The UCNP has a core of NaYF_4_:Yb/Er, enclosed in an organic silica shell with a rattle structure, and then sequentially modified with photosensitizer and Rose Bengal (RB). When illuminated by NIR light, the core of UCNP can efficiently absorb light energy and emit green light, which can excite RB to produce ROS. And the structure of Aβ42 can be effectively disrupted by ROS (see Figure 12A). As shown in Figures 12B–E, under the irradiation of near-infrared light, Aβ monomers co-incubated with NaYF_4_:Yb/Er@SiO_2_@RB cannot aggregate into Aβ peptide, and their toxicity is also effectively reduced (Zhang et al., 2019). However, the above-mentioned UCNPs lack selectivity for Aβ aggregates, thus limiting their application *in vivo* research. To overcome this problem, Du et al. designed and developed an upconversion nanoprobe UCNP@C_60_-pep with targeting Aβ plaques (Du et al., 2018). UCNP@C_60_-pep, which is characterized by its abundant fullerene structure, combined with KLVFF, a kind of targeting peptide. Due to the C_60_ fullerene structure rich in π double bonds, it has the ability to generate and quench ROS. These two opposing properties of C_60_ can be modulated by NIR light, allowing it to play a synergistic role in AD therapy. UCNP@C_60_-pep can generate ROS under NIR light irradiation, causing the dissociation of Aβ aggregates. Subsequently, the hydrophilic oxygen atoms combine with the hydrophobic Aβ to prevent the accumulation of toxic plaques. However, in the absence of NIR light, UCNP@C_60_-pep can quench excessive ROS to ensure the redox balance of the intracellular environment. The results of immunofluorescence labeling experiments show that UCNP@C_60_-pep can inhibit the aggregation of Aβ and reduce the expression of Aβ_42_ that can cause paralysis of *Caenorhabditis elegans* (CL2006) strain under NIR light irradiation (Figures 12F–J).

### 4.3 Photothermal therapy

In addition, PTT, always known for its high-effectiveness therapy of tumors (Wen et al., 2020), also serve as an effective technique to interfere with Aβ aggregation. The hyperthermia induced by the photothermal effect can destabilize physiological conditions that are crucial for the formation of Aβ aggregation (Stine et al., 2003). In addition, local heating can cause an increase in heat shock proteins, thereby promoting the refolding of aggregated proteins (Czarnecka et al., 2006).

**Figure 13:**
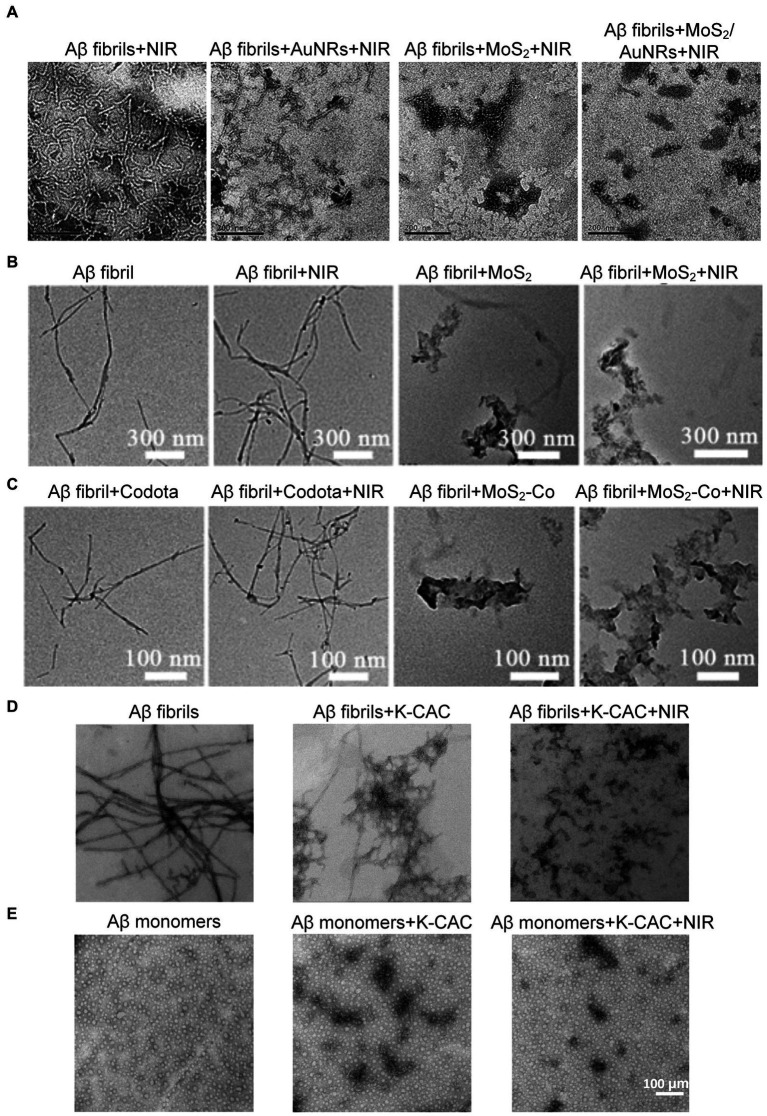
Studies on degradation of Aβ deposits using photothermal nanoprobes. **(A)** TEM images of the Aβ fibrils alone and co-incubated with AuNRs, MoS_2_, and MoS_2_/AuNRs. All groups were irradiated by NIR laser for 10 min Reprinted with permission from Wang et al. (2019). Copyright 2019 Royal Society of Chemistry. Scalebar is 200 nm. **(B,C)** Are the changes of Aβ fibrils morphology after various treatments. Reprinted with permission from Ma et al. (2019). Copyright 2019 Wiley-VCH. **(D)** TEM images of Aβ fibrils only and co-incubated with K-CAC under NIR laser irradiation. **(E)** TEM images of Aβ monomers only and co-incubated with K-CAC under NIR laser irradiation for 7 days. Reprinted with permission from Ge et al. (2022). Copyright 2022 American Chemical Society.

Considering high photothermal conversion efficiency (PCE) is a vital characteristic of photothermal agents, Wang et al. designed multifunctional MoS_2_/AuNRs nanocomposites with relatively high PCE, by incorporating the two materials together, each with optimal properties for the degradation of Aβ fibrils under low-power NIR laser irradiation (Wang et al., 2019). Figure 13A shows TEM images of Aβ fibrils treated with different conditions, including AuNRs, MoS_2_, and MoS_2_/AuNRs. All groups were irradiated with NIR laser for 10 min. It is obvious that only treated with NIR laser irradiation cannot influence the structure of Aβ fibrils which were co-incubated without nanomaterials. However, Aβ fibrils co-incubated AuNRs or MoS_2_ showed a large number of small fibrils and amorphous aggregates were observed after irradiating. More importantly, samples treated with MoS_2_/AuNRs displayed the shortest fibrils after NIR laser irradiation. These results directly illustrated the degradation ability of MoS_2_/AuNRs, which brings the possibility for treating AD.

Since the cleavage site of Aβ is embedded within its structure, which hinders the application of cleavage enzymes, Ma et al. combined two-dimensional (2D) nanosheets MoS_2_ with 1,4,7,10-tetraazacyclododecane-1,4, 7,10-tetraacetic acid (Co-DOTA), MoS_2_-Co was prepared, which is an artificial enzyme activated by NIR light (Ma et al., 2019). MoS_2_-Co possesses notable stability and are low cost in synthesis, which has greatly promoted the development of artificial Aβ-degrading enzymes to replace natural enzymes. It is special that MoS_2_ − Co can not only degrade Aβ monomers under NIR light irradiation, but also produce thermal effects, leading to the degradation of Aβ aggregates (Figures 13B,C). What’ more, the photothermal effect can significantly enhance the permeability of the BBB, making it easier for nanomedicines to reach Aβ deposition sites. Ge et al. combined the oxidation properties of ceria nanoparticles (CeO_2_) to design a nanoprobe for combined photothermal and photodynamic treatment of AD (Ge et al., 2022). In addition, in order to better hinder the self-assembly of Aβ, the middle surface of the probe was modified with KLVFF, a pentapeptide fragment that can bind to the Aβ_16-20_ region, preventing the Aβ monomers from becoming fibrils (Li M. et al., 2017; Xiong et al., 2017; Zhao et al., 2019). As shown in Figure 13D, Aβ fibrils showed an elongated branched structure, and the size of Aβ fibrils was significantly reduced after co-incubation with K-CAC. More importantly, after NIR laser irradiation, the fiber structure was almost completely destroyed, demonstrating the probe’s ability to efficiently degrade AB fibrils under light. In contrast, Aβ monomers co-incubated with K-CAC were unable to aggregate and were effectively decomposed regardless of light exposure (see Figure 13E).

## 5 Conclusion and perspectives

Upon this review, we present a narrative review discussing the utilization of PA outcomes in both the diagnosis and treatment of AD. Specifically, regarding diagnosis, we explore the application of PA imaging—an innovative noninvasive technique that combines the benefits of optical contrast with ultrasonic detection. The manuscript begins by delineating the operational mechanism of PA imaging and its ongoing exploration in cancer research and neurological studies. The application of PA in the diagnosis and assessment of AD status is then described, including structural alterations, functional parameters, and molecular details. Biomedical imaging can help identify structural alterations in Aβ that can differentiate the AD-affected brain from the healthy brain. In addition, damage to the meningeal lymphatics can also adversely affect brain function. Functional parameters, such as vascularity, oxygen saturation, hemoglobin concentration, undergo substantial alterations, even in the initial stages of AD, contributing to alterations in the optical spectrum. Therefore, PA imaging can provide a feasible approach for early diagnosis and assessment of the developmental stages of AD. Additionally, PA imaging provides the possibility to detect and quantify changes in molecular information. For instance, it can measure Cu^2+^ concentration, an important indicator of oxidative stress levels in the brain, using customized nanoprobes. In summary, by incorporating structural, functional, and molecular imaging abilities, PA imaging manifests the potential as a tool for timely sensing and treatment of AD.

In this review, we explore PA cavitation therapy, PDT, and PTT for treating AD. PA cavitation takes advantage of strong physical effects and effectively disrupts protein structure, providing a promising approach for therapy. Although PDT and PTT are widely used in cancer therapy, their use in the treatment of AD is still limited. However, their noninvasive nature and proven efficacy suggest that they have significant potential in AD treatment. Furthermore, the utilization of molecular-targeted contrast agents could further enhance the precision, efficacy, and recovery outcomes of treatment. In fact, clinical trials of optical methods in the diagnosis or treatment of AD are currently limited to superficial penetration depths of the human brain, focusing mainly on animal models. Striking a balance between imaging depth and resolution is a major challenge. Simultaneously achieving high-resolved and deep-penetrated PA imaging is the key to its clinical promotion. This advancement holds profound significance for the clinical diagnosis of AD and other diseases.

Absolutely, in addition to the imaging depth, enhancing the performance of current nanoprobes is essential for improving PA detection sensitivity and discrimination. Nowadays, the nanoprobes utilized for PA or photothermal contrasts typically rely on changes in permeability or microenvironment to passively bind to AD regions. It is necessary to innovate probes that actively target specific AD markers to improve the effectiveness for diagnosis and treatment. Indeed, nanoparticles might accumulate in adjacent regions, thereby potentially mitigating the contrast and efficiency for both PA diagnosis and photothermal-triggered treatment. Unintentional aggregation of nanoparticles may undermine the accuracy and effectiveness of these methods in precisely targeting specific regions affected by AD. Identifying pivotal molecules associate with early-stage AD and creating targeted probes could empower PA to precisely locate early AD lesions, thereby significantly enhancing treatment efficiency through photothermal trigger without damages to nearby healthy tissues. Given the common photothermal mechanism of PA and PTT, combining these two modules into a system capable of simultaneously detecting PA and early AD and timely PTT treatment is a feasible approach, providing a promising treatment option for AD. Despite that there have been notable efforts to utilize PA for AD imaging, most studies are confined to laboratory proof-of-concept. While nano-contrast agents for PA molecular imaging contribute to AD diagnosis, various concerns must be taken into consideration before clinical translation. These include considerations regarding their antigenicity, noxiousness, particle dimensions, and other factors.

In summary, PA imaging provides high-resolved and high-contrast imaging for tissue structures, enabling the identification of specific tissue components. Moreover, its combination with molecular nanorobotics shows great clinical market potential for targeted imaging. This technology has important economic and social significance, which promotes the utilization of PA imaging in clinical application and industrialization. This mini-review is aimed at enhancing the comprehension of AD diagnosis and treatment through breakthrough innovation of tissue photothermal effect. It is hoped to inspire further exploration in this field for more effective and earlier diagnosis and treatment of AD.

## Author contributions

JM: Conceptualization, Data curation, Formal analysis, Resources, Validation, Writing – original draft, Writing – review & editing, Investigation, Methodology, Project administration, Software, Supervision, Visualization. CL: Conceptualization, Data curation, Formal analysis, Funding acquisition, Investigation, Methodology, Project administration, Resources, Software, Supervision, Validation, Visualization, Writing – original draft, Writing – review & editing. HC: Supervision, Validation, Writing – review & editing. YQ: Conceptualization, Methodology, Supervision, Validation, Writing – review & editing. JZ: Formal analysis, Methodology, Project administration, Validation, Writing – review & editing. YZ: Methodology, Resources, Supervision, Validation, Visualization, Writing – review & editing. YL: Conceptualization, Investigation, Project administration, Supervision, Writing – review & editing. LW: Investigation, Supervision, Validation, Visualization, Writing – review & editing. DT: Conceptualization, Data curation, Methodology, Supervision, Validation, Writing – review & editing.
